# An Industrial-Grade Solution for Crop Disease Image Detection Tasks

**DOI:** 10.3389/fpls.2022.921057

**Published:** 2022-06-27

**Authors:** Guowei Dai, Jingchao Fan

**Affiliations:** ^1^National Agriculture Science Data Center, Agricultural Information Institute, Chinese Academy of Agricultural Sciences, Beijing, China; ^2^National Nanfan Research Institute (Sanya), Chinese Academy of Agricultural Sciences, Sanya, China

**Keywords:** crop disease detection, convolutional neural network, model compression, knowledge distillation, activate quantitative, model deployment

## Abstract

Crop leaf diseases can reflect the current health status of the crop, and the rapid and automatic detection of field diseases has become one of the difficulties in the process of industrialization of agriculture. In the widespread application of various machine learning techniques, recognition time consumption and accuracy remain the main challenges in moving agriculture toward industrialization. This article proposes a novel network architecture called YOLO V5-CAcT to identify crop diseases. The fast and efficient lightweight YOLO V5 is chosen as the base network. Repeated Augmentation, FocalLoss, and SmoothBCE strategies improve the model robustness and combat the positive and negative sample ratio imbalance problem. Early Stopping is used to improve the convergence of the model. We use two technical routes of model pruning, knowledge distillation and memory activation parameter compression ActNN for model training and identification under different hardware conditions. Finally, we use simplified operators with INT8 quantization for further optimization and deployment in the deep learning inference platform NCNN to form an industrial-grade solution. In addition, some samples from the Plant Village and AI Challenger datasets were applied to build our dataset. The average recognition accuracy of 94.24% was achieved in images of 59 crop disease categories for 10 crop species, with an average inference time of 1.563 ms per sample and model size of only 2 MB, reducing the model size by 88% and the inference time by 72% compared with the original model, with significant performance advantages. Therefore, this study can provide a solid theoretical basis for solving the common problems in current agricultural disease image detection. At the same time, the advantages in terms of accuracy and computational cost can meet the needs of agricultural industrialization.

## Introduction

Crop disease assessment is necessary for the agricultural industry to understand crop quality and yield levels. Many factors affect crop yield, and it is generally accepted that crop yield varies from year to year depending on changes in climate, soil parameters, and fertilizers used. With the introduction of precision agriculture (Cisternas et al., 2020), crop life cycle processes, such as sowing, monitoring, weed control, pest and disease management, and harvesting also positively impact crop yield. Crop diseases affect leaves, stems, roots, and fruits, limiting crop growth and development and thus affecting crop quality and yield. World crop yields are estimated to be reduced by 11–30% annually due to crop diseases and pests (Deng et al., 2021). The leading causes of the emergence of these diseases are microbial, genetic diseases, and diseases caused by infectious agents, such as bacteria, fungi, and viruses. Secondary factors contributing to diseases are rainfall, humidity, temperature, and nutrient deficiencies.

There are many traditional methods to diagnose crop diseases. The most direct method is the human visual estimation, this method crop disease diagnostic technique relies on farmers' experience, and the corresponding expert system requires the writing of a large number of calibration rules, which is time-consuming and limited by the expert's empirical knowledge and has a limited scope of application. In contrast to traditional crop disease diagnostic techniques, some challenging, expensive, and time-consuming methods that require highly specialized operations have been proposed, one using spectroscopy to diagnose whether crop leaves are healthy and infected (Sanchez et al., 2020) and another method using polymerase chain reaction (Urbina et al., 2021) to extract DNA from leaves and analyze key fragments of DNA to determine whether crop leaves are healthy or infected. With the rapid development of artificial intelligence to promote precision agriculture, some fast and efficient AI detection methods (Jiang H. et al., 2020; Su et al., 2021; Tulbure et al., 2022) have been proposed to enable the development of automatic crop disease detection techniques through recent advances in artificial intelligence (AI), machine learning (ML), and computer vision (CV) technologies that are time-sensitive and efficient enough to accurately detect crop leaf diseases without human intervention. The application of artificial intelligence techniques in agriculture (Sharma, 2021; Dewi et al., 2022; Figueroa-Mata et al., 2022; Walker et al., 2022; Zhao et al., 2022) has made it essential to address various challenges of agricultural products, such as environmental impact, productivity, food security, and sustainability, by using new types of methods to solve many of the problems faced by farmers in the past. The strong applicability to the problems makes it easy to solve compound problems.

Current research related to plant disease detection in computer vision is divided into two main categories: methods based on manual features and in-depth learning features. Most of the existing studies belong to the former category (Chen et al., 2020; Jiang F. et al., 2020; Dawod and Dobre, 2022), which identifies objects in the feature space using manually extracted features as localizers or classifiers. Manual features have the advantage of localization and simplicity. However, they may lack the ability to extract the semantics and discriminate features in a changing environment and usually select appropriate features based on experience. Deep learning models solve the problem of manual feature extraction and are therefore widely used in various applications of crop disease measurement (Lawal, 2021; Li et al., 2021; Wani et al., 2022) Deep learning-based plant disease detection networks can be divided into the following networks: two-stage networks represented by Faster region-based convolutional neural network (Faster R-CNN) (Ren et al., 2017); one-stage networks represented by Single Shot Multibox Detector (SSD) (Liu et al., 2016), and You Only Look Once (YOLO) (Redmon and Farhadi, 2016, 2018; Redmon et al., 2017; Bochkovskiy et al., 2020). The main difference between the two networks is that the two-stage network needs first to generate a candidate frame (Proposal) that may contain lesions before performing the target detection process. In contrast, the one-stage network directly uses the features extracted from the network to predict the location and class of lesions. In agriculture, a one-stage network has apparent advantages over a two-stage network. The network represented by YOLO has the most advanced performance in target detection, with higher computational speed and better computational efficiency. YOLO (Redmon et al., 2017) combines the region proposal network (RPN) branching and classification stages in a single network, making its architecture more concise, and the YOLO model predicts the bounding boxes and their corresponding classes directly through a feedforward network compared to the previous region proposal-based detectors (Ren et al., 2017). YOLOV2 is the second version of YOLO; introducing anchors in YOLO V2 (Redmon and Farhadi, 2016) was inspired by Faster R-CNN; anchors improve the detection accuracy and simplify the learning process of the problem and the network. YOLO and YOLO V2 are the foundations of YOLO V3 (Redmon and Farhadi, 2018). YOLO V3 employs multi-label classification, in which each label calculates the classification loss using binary cross-entropy loss rather than mean square error, predicts objects at three different scales, and uses logistic regression to predict the score of each bounding box. YOLO V4 (Bochkovskiy et al., 2020), the next version of YOLO V3, consists of CSPDarkNet53 as the backbone, SPP (Spatial Pyramid Pool) as an additional block, Path Aggregation Network (PANet) as the neck, and YOLO V3 head together to improve the training accuracy by introducing new methods of data enhancement, optimized hyperparameters, and genetic algorithms.

Afzaal et al. (2021) reported the studies obtained using classical convolutional neural networks, namely GoogleNet, VGGNet, and EfficientNet, to identify potato leaf diseases at different growth stages. Sharma et al. (2021) proposed a CNN model for rice and potato leaf disease classification, which was able to classify rice images and potato leaves with 99.58% accuracy, outperforming other advanced machine learning image classifiers, such as SVM, KNN, decision trees, and random forests. To demonstrate the feasibility of deep learning algorithms based on an encoder-decoder architecture for semantic segmentation of potato late blight spots based on field images, Gao et al. (2021) used a SegNet-based encoder-decoder neural network architecture for lesion segmentation, which can extract semantic features from low to high level, in a disease test dataset with leaves and soil in the background to intersect and union (IOU) values of 0.996 and 0.386, respectively. Rashid et al. (2021) proposed a multilevel deep learning model to classify potato leaf diseases called PDDCNN. First, potato leaves were extracted using the YOLOV5 image segmentation technique from potato plant images. Then early blight and late blight of potato were classified by PDDCNN, which also used data enhancement techniques to improve the accuracy. Finally, the final accuracy was 99.75%. Mathew and Mahesh (2022) detected bacterial spot disease in sweet pepper plants by YOLOV5 and the training time was only 9.5% of the YOLO V4 model for the same accuracy. Zhao et al. (2021) extracted 10 classes of tomato leaf diseases from the PlantVillage dataset for training for multiple plant disease identification. They established the SE-ResNet50 model by embedding the attention mechanism SENet module into ResNet50, which achieved average recognition accuracy of 96.81% on the tomato leaf disease dataset.

Analyzing the above research process, the identification of crop diseases is mainly divided into image processing, texture feature extraction of crops, inputting machine learning for detection, or using convolutional neural networks for deep crop feature identification and extraction. However, the above studies have made good progress in crop image detection. However, related research is still only at the theory, exploration, and introduction stage. It is mainly because most of them only consider the accuracy of a single scene dataset and ignore the storage size, inference time, deployment cost, and application environment that need to be considered in the actual production of the model. Specifically, they are divided into the following deficiencies:

1. High computational cost: With the continuous development of neural networks, image detection tasks require a large and complex network with a large number of parameters to achieve higher accuracy. Typically, training a sizeable parametric network model will require mighty computer power and data storage capacity. However, the prohibitive computational cost and memory greatly hinder the deployment of CNNs on limited platforms with a wide range of resources, especially for frequently executed tasks or real-time applications. For agricultural application scenarios, the focus should be on requirements limited by the natural environment in the field and low-cost deployment, and simplicity of use.
2. Low generality of methods: Existing studies usually extract relevant data from the PlantVillage dataset, which are too old and unbalanced in terms of categories, covering fewer disease categories. On the other hand, most methods do not evaluate the performance of images with more crop categories and different severity of the same disease. This is because fewer disease categories are detected, coupled with features that are easier to distinguish. When tested on fewer categories of diseased leaves, any model version can be marked as good.
3. Long training period: when deep learning models are put into production environments, the use of classical neural network models or the use of two-stage (Duan et al., 2020) class models in training on datasets, due to their large number of model parameters, or due to the need to calculate Region Proposal first, and the backpropagation calculation is slow, and the development cost is too high, maintenance and scaling difficulties, it is difficult to be mobile device deployment.

This study solves the above problem and proposes a feasible technical solution. The significant contributions of this manuscript are as follows:

1. Model acceleration: The YOLO V5 model in one-stage was used as the base. Model accuracy is maintained by merging model pruning and knowledge distillation to make the model lighter while keeping model accuracy, considering the importance of model parameter size for the training environment of agricultural application scenarios. Activation Compressed Training Neural Network (ActNN) is chosen to perform dynamic random parameter quantization of YOLO V5 models to realize training tests of large parameter models when device memory is insufficient, thus ensuring comprehensive performance in different device environments. The model is characterized by high recognition accuracy and fast inference.
2. Model compression: The goal is to use various recomputation methods of CNN models to accelerate model inference, compress model parameters, intermediate activation results, and optimizer states, and minimize model storage space without severely compromising detection accuracy.
3. Model generalization: Extensive use of multiple publicly available data sets to build models for detecting multiple crop diseases and disease severity. Cover the need for a single model to detect multiple diseases to meet the standards of industrial-grade applications. Address the insufficient number of crop disease samples and category imbalance in public datasets by using datasets to fuse features, balance categories, and reduce differences due to multiple factors, such as shape, variety, and environmental factors.
4. Model deployment: To integrate multiple platforms and consider the specificity of model deployment in agricultural applications, the models are converted to Open Neural Network Exchange (ONNX) format. The CNN model forward computation is accelerated by importing a suitable framework to achieve efficient and stable deployment.

We built and optimized the datasets in this article on three representative datasets to demonstrate that this article's research could cover most agricultural disease image recognition scenarios. Our study fully considered the issues of model storage size, inference time, deployment cost, and application environment, and integrates the state-of-the-art YOLO V5 with these technologies for the first time, which is the innovation of this article.

This section summarizes issues relevant to this study and briefly describes related research. The remainder of the article is structured as follows: section Materials and Methods describes the material used in the article, and it is primarily concerned with the methodology. Section Experiment describes the model training equipment environment and parameters, and several experiments are fully implemented using the methods described in section Materials and Methods, with the results analyzed and discussed. Finally, section Conclusion summarizes the main conclusions and contributions of this work.

## Materials and Methods

**Figure 1:**
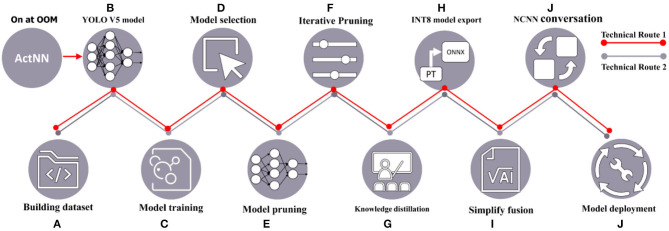
Technical route diagram.

The technical route of the industrial-grade crop disease image detection task solution proposed in this article is shown in Figure 1. The crop disease dataset labeled by plant pathologists is inputted into the YOLO V5-CAcT model for training, and the best model is selected to achieve rapid recognition of the target the model. Considering the importance of inference time and model size for almost all agricultural application scenarios, two model compression technology routes are proposed, with the red line in Figure 1 indicating technology route 1 and the gray line indicating technology route 2. The two technical routes can be used in combination in different environments. Technical route 1 is generally an adjunct to technical route 2 and can be adapted to any different equipment environment for training tests. For the sake of description, technology route 1 is not combined with technology route 2. The two technology routes have overlapping parts, and Figure 1b ActNN changes some of the original modules in YOLO V5 to take on the tasks that follow from Figure 1b. Technique 1 uses ActNN to randomly quantization the YOLO V5 model to reduce memory consumption when memory is insufficient. Technical route 2 uses model pruning and knowledge distillation to optimize model parameters, thus simplifying the structure and parameters of the model while maintaining accuracy.

### Based on YOLOV5 Crop Disease Detection Technology

**Figure 2:**
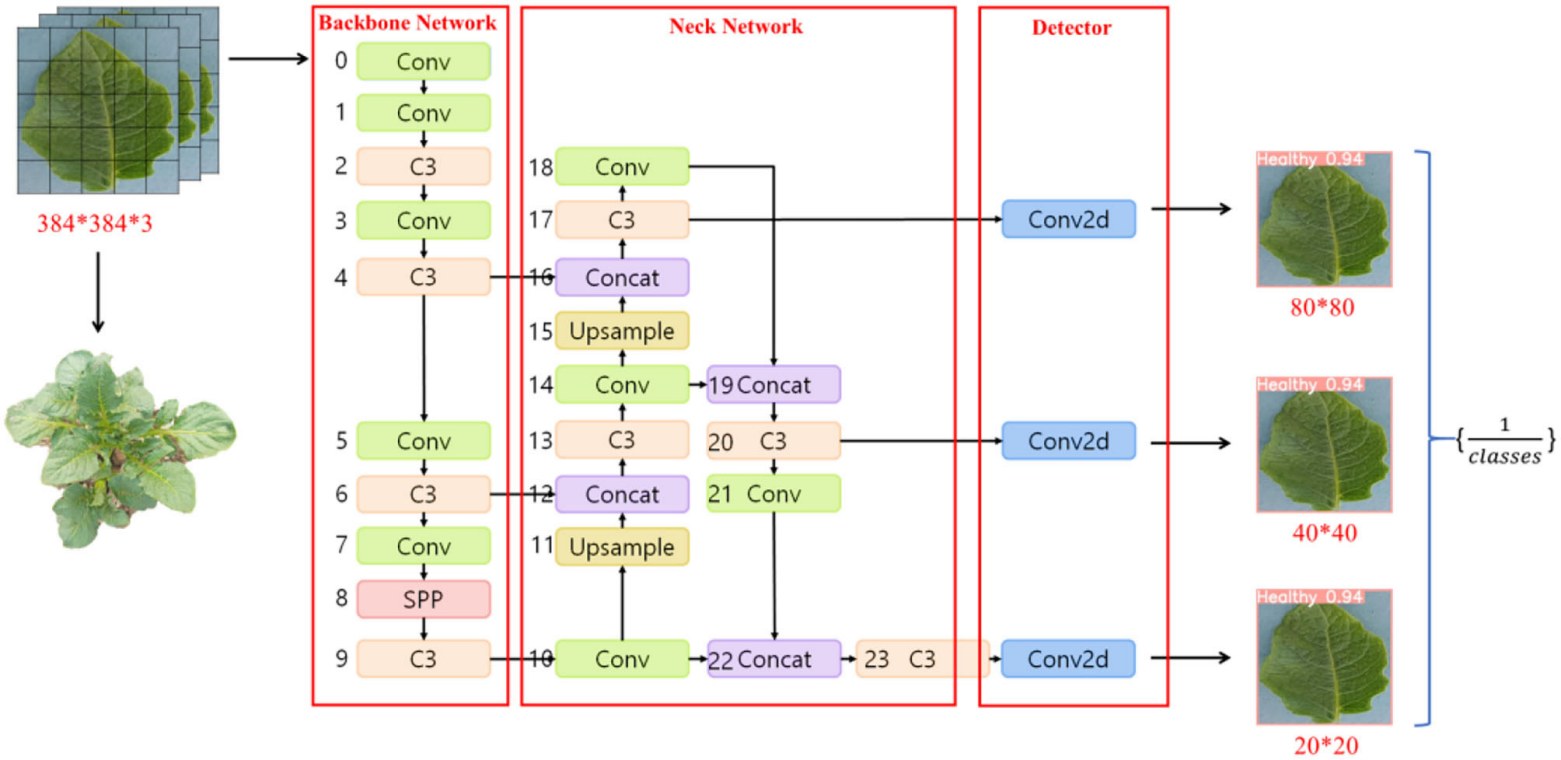
Crop disease YOLOV5 network structure diagram.

You Only Look Once is the most representative target detection algorithm in the One-Stage family of algorithms, and the latest product of the YOLO architecture family is the YOLO V5 network (Jocher et al., 2021). This model has high recognition accuracy fast inference speed and avoids the candidate region recomputation in a two-stage algorithm. So far, the YOLO V5 algorithm has been iterated for six versions. Each version is launched, representing the latest technology in target detection, with features suitable for promotion in precision agriculture. Again, the YOLO V5 target recognition network model has a smaller weight file, nearly 90% smaller than YOLO V4 (Yan et al., 2021), which indicates that the YOLO V5 model is suitable for deployment to embedded devices instantaneous detection. Thus, the advantages of the YOLO V5 network are high detection accuracy, lightweight attributes, and fast recognition speed. The YOLO V5 architecture contains four main structures named YOLO V5l (Yan et al., 2021), YOLO V5x (Yan et al., 2021), YOLOV5m (Yan et al., 2021), and YOLO V5s (Yan et al., 2021), which have a decreasing number of model parameters in order. To adapt the mobile solution, the YOLO V5n(Nano) model is later proposed, which has the same model depth, reduced network width from 0.5 to 0.25, and reduced model parameters by 5.6 M compared to YOLO V5s, and derives the model at INT8 accuracy, which is only 2.1 MB in size. In this article, YOLOV5n(Nano), YOLO V5s, YOLO V5m, and YOLO V5l are used as benchmark test models, as shown in Figure 2.

The YOLO V5 framework consists of three main structures, including Neck Network, Backbone Network, and Detect Network. Neck Network is a convolutional neural network that combines fine-grained images and forms image features. Precisely, Neck Network aims to reduce the computation of the model and speed up the training. The Conv module is the basic convolutional YOLO V5, which performs two-dimensional convolution, two-dimensional regularization, and weighted linear unit (SiLU) activation (Singla et al., 2021) operations on the input in turn. C3 module consists of 3 Conv with many Bottlenecks, in which the structure of the composition is added to the calculation map in turn. Bottleneck completes the residual feature transfer without reducing the features. Moreover, the output results in Concat stitching, the output depth is the same as the input depth. C3 module converts the input data, calculates in the Bottleneck layer, adds the initial input Conv value and the calculated value of Bottleneck in Concat, and converges and outputs. Bottleneck continues to process Conv(1, 1) on the input value and outputs the calculated value of Conv(3, 1). after the Conv operation, SPP performs a Max Pooling operation using 5^*^5, 9^*^9, and 13^*^13 to combine the three Max Pooling values in Concat with the Conv values in the current input value, and Conv is sent after Conv. Upsample is a Pytorch base library function that doubles the number of each feature mapping array in the structure values; Concat plays the role of merging input layers.

The eleventh and fifteenth layers of Neck Network use Upsample module to expand the features, and the features extracted from the four and sixth layers of Backbone Network are passed to Neck Network for fusion. The fourth layer of the Neck is further fused with the Upsampled fifteenth layer using Concat. Then the fourteenth fused layer is fused with the second After the fusion, the fourteenth layer is fused with the eighteenth layer again. The small target detection uses the deeper ninth layer network features fused with the twenty-first layer Conv, and the fusion forms a larger fixed feature map output to the Detector for prediction. Since Detector currently has three Conv2d values, the three values are combined and output. YOLO V5 has three feature detection scales for feature detection of different sizes, while YOLO V5 has the feature of enhanced training data; the data loader can perform many types of data augmentation.

### Model Compression and Deployment

With the depth and complexity of neural network architecture, the computation required to train State Of The Art (SOTA) AI models (Gholami et al., 2021) is growing at a rate of 15 times every 2 years, and the number of parameters of large Transformer models is growing exponentially at a rate of 240 times every 2 years (Gholami et al., 2021); the breakthrough of deep learning performance cannot be achieved without the crazy growth of the model size, and models with a more significant number of parameters usually have better performance It has become an industry consensus; however, the resulting memory wall problem limits the exponential growth of AI model parameters; therefore, a combination of model compression and hardware systems is usually required to optimize the structure of CNN models to achieve better maintenance performance. Model compression is a technical solution to address this problem during the model training phase by simplifying the model structure by reducing redundant parameters and speeding up model inference without significantly degrading performance. Current research on model compression techniques includes neural network pruning, low-precision quantization, knowledge distillation, and activation weight compression.

#### Neural Network Pruning

There are usually significant, redundant parameters between deep neural network model layers. Some of them play a feeble role in the target detection process, and the cumulative impact of these parameters on the feature map is negligible, and removing these parameters has little impact on the accuracy of target detection; therefore, the parameters between model layers need to be further compressed and optimized. Model pruning is a widely used model compression technique, and from the perspective of pruning granularity, pruning methods can be classified as structured and unstructured pruning (Wang et al., 2021), Filter Pruning *via* Geometric Median (FPGM) (He et al., 2019) is a structured weight pruning. The essence of the algorithm is to identify the geometric median close filters present in the network and achieve the purpose of streamlining the weights to accelerate inference by eliminating the redundant filters and their associated input-output relations. The geometric median is calculated as shown in Equation (1).

(1) $$\begin{array}{r} x^{*}=\underset{x\in {\mathbb{R}}^{d}}{\arg min}\;f\left(x\right)\;where\;f\left(x\right)\underset{i\in \left[1,n\right]}{\sum }∥{x-a^{\left(i\right)}∥}_{2} \end{array}$$

where x^*^ is the minimum value of the parameter in d-dimensional space, denoting the geometric median; *f*(*x*) is the minimum value of the sum of Euclidean distances from *N* points *a*^1^ to *a*^*i*^, each *a*^*i*^ ∈ ℝ^*d*^; Equation (2) uses the geometric median of Equation (1) to obtain the sum of Euclidean distances of all filters in layer *i*;

(2) $$\begin{array}{r} g\left(x\right)=\underset{j^{\prime }\in \left[1,N_{i+1}\right]}{\sum }∥{x-F_{i,j}^{\prime }∥}_{2}\;x\in R^{N_{i}\times K\times K} \end{array}$$

$F_{i,j}^{\prime }$ denotes the filters in layer *i*, *x* is the tensor of layer *i*, $x\in \left\{F_{i,1},\ldots ,F_{i,N_{i+1}}\right\}$; Equation (3) $F_{i}^{GM}$ denotes the geometric median of layer *i*. The sum of the Euclidean distances of all filters in *g*(*x*) is substituted into Equation (3) to obtain the smallest geometric median within layer *i*. This median denotes the data center of the layer.

(3) $$\begin{array}{r} F_{i}^{GM}\in \underset{x\in {\mathbb{R}}^{N_{i}}\times K\times K}{\arg min}g\left(x\right) \end{array}$$

If we consider the existence of filters close to the geometric median in layer *i* is redundant, it can be considered that this filter is replaceable, and the $F_{i,j^{*}}$ calculated in Equation (4) indicates the proximity of replaceable filters, and the proximity region of the replaced network has little impact on the whole network. Therefore, replaceable filters are determined for all layers $F_{i,j^{*}}$ of the network model.

(4) $$\begin{array}{r} F_{i,j^{*}}\in \underset{j^{\prime }\in \left[1,N_{i+1}\right]}{\arg min}{∥F_{i,j^{\prime }}-F_{i}^{GM}∥}_{2} \end{array}$$

Equations (2) and (4) can be further expressed as Equation (5). From Equation (4), we can see that $F_{i,j^{*}}$ can be replaced as $x-F_{i,j^{*}}=0$, then *g*′(*x*) = *g*(*x*), thus, cutting these redundant filters can further reduce the model.

(5) $$\begin{array}{l} \begin{array}{c} g^{\prime }\left(x\right)=g\left(x\right)-\sum_{j^{\prime }=j^{*}}∥{x-F_{i,j^{\prime }}∥}_{2}\\ \;=g\left(x\right)-{∥x-F_{i,j^{*}}∥}_{2} \end{array} \end{array}$$

**Figure 3:**
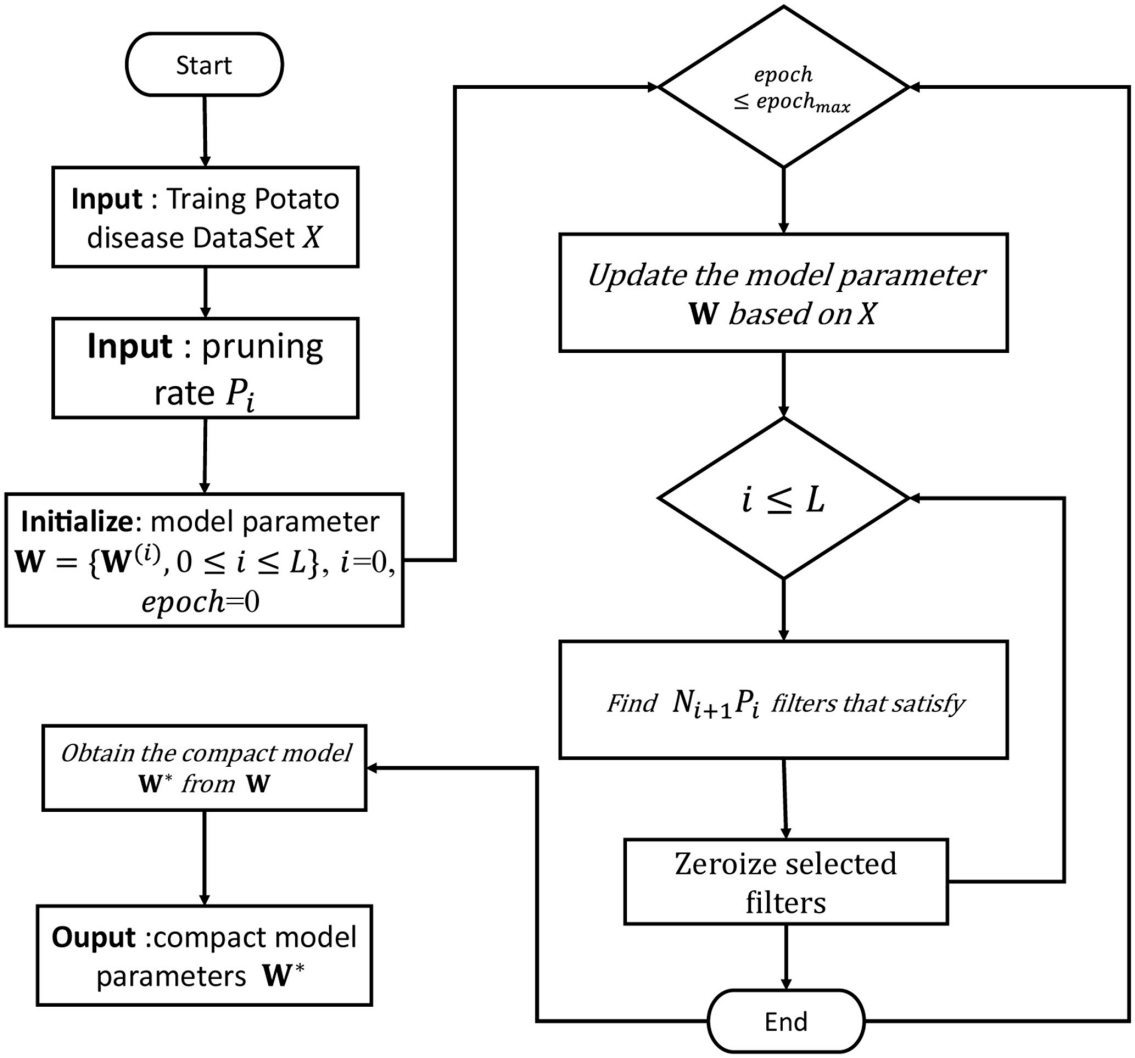
FPGM pruning process.

After FPGM pruning and then iterative pruning, the network can be quickly restored to its original performance. AutoSlim, an open-source automated model pruning tool (wzx, 2021), divides the model pruning function into three major architectures and supports authors to package their own SOTA pruning algorithms. Based on this, this article constructs a pruning algorithm supporting YOLO V5 and implements its FPGM-YOLOV5 algorithm. The FPGM-YOLOV5 pruning process is summarized in Figure 3.

#### Knowledge Distillation

After model pruning, the accuracy of the model generally decreases. Even if the pruned model is fine-tuned again, the accuracy may still have a large gap with the model before pruning. Therefore, this article can solve this problem by minimizing the accuracy loss by Knowledge Distillation (KD). Knowledge distillation uses transfer learning to supplement specific parameters missing in the small model to achieve the recognition accuracy of the large model as much as possible. Knowledge distillation can be regarded as a model compression method, where the large model is the teacher and the miniature model is the student.

Usually, the traditional training process finds the excellent likelihood for the ground truth under Hard Label. In contrast, the training process of KD uses the category probabilities of the teacher model as soft targets (Labels With Probabilities) to guide the training of the student model. The knowledge describing the similarity of different categories of information can be transferred from these soft targets (Hinton et al., 2015) to improve the performance of the student model.

**Figure 4:**
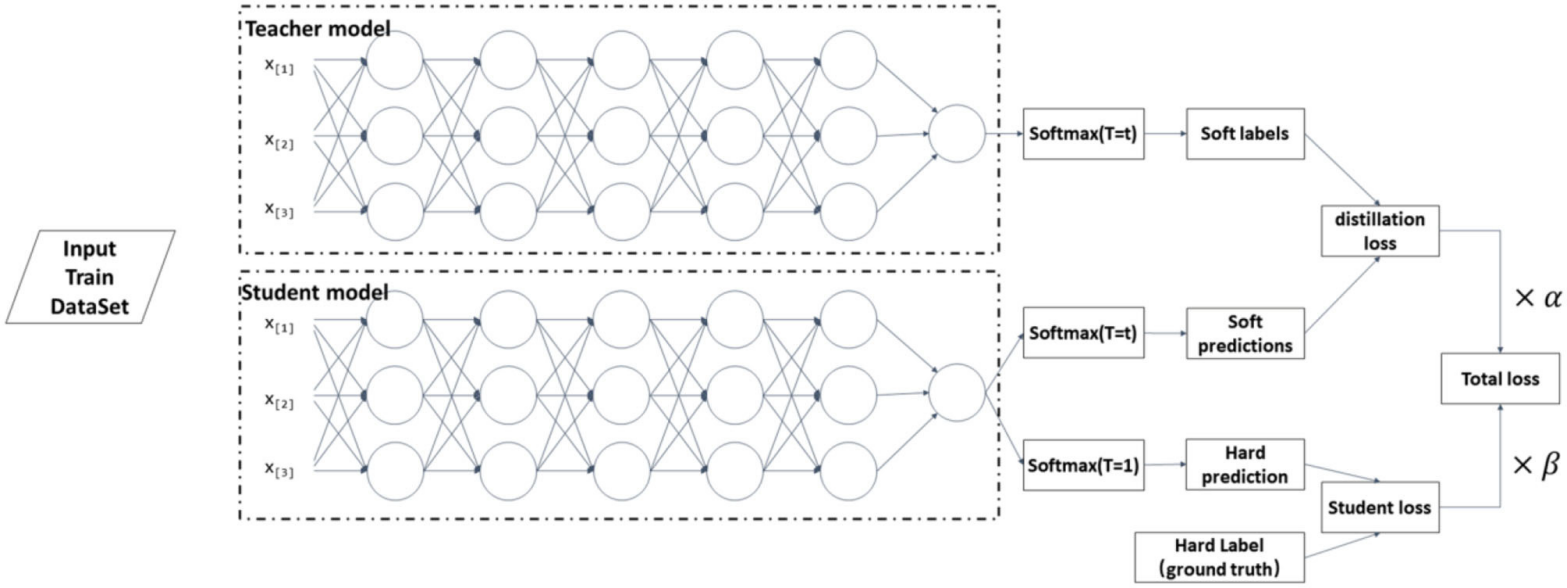
Step of knowledge distillation.

Figure 4 shows the primary technical process of knowledge distillation. The teacher model is the original model with high training accuracy in the knowledge extraction process. The pruned original model is the student model, with a small number of parameters and a relatively simple model structure. The teacher model uses a series of hyperparameters to converge to the optimal state according to the established principles. Then, the same hyperparameters of the teacher model are used to train the student model for knowledge distillation. The distillation loss is corrected by coefficients β for the distillation loss of the teacher model and the student model where the Hard Label (Ground Truth) can effectively reduce the possibility of errors being propagated to the student model. Measuring the similarity of student and teacher models can be expressed in Equation (6), $L_{R}$ is a function that can measure the similarity, expressed explicitly in *softmax*. In general, when the entropy value of the probability distribution output from *softmax*

(6) $$\begin{array}{r} L_{ResD}\left(z_{t},z_{s}\right)=L_{R}\left(z_{t},z_{s}\right) \end{array}$$

(7) $$\begin{array}{r} softmax\left(I,T\right)=\frac{\exp\left(I/T\right)}{\sum_{i}\exp\left(I_{i}/T\right)} \end{array}$$

is relatively small, the value of negative labels is very close to 0, which contributes very little to the loss function, which leads to a reduction in the attention of the student model for negative labels during distillation, which is addressed by the temperature coefficient T in Equation (7). Where *I* is the logits input to the *softmax* layer, the higher *T*, the more the *softmax* output category value probability flat. The total loss *L*_*total*_ is represented by Equations (8)–(10), α and β are equilibrium coefficients, *L*_*soft*_ is distillation loss, and *L*_*hard*_ is student loss; in *L*_*soft*_, *N* is the number of labels,

(8) $$\begin{array}{r} L_{total}=\alpha L_{soft}+\beta L_{hard} \end{array}$$

(9) $$\begin{array}{r} \{\begin{array}{c} L_{soft}=-\sum_{j}^{N}p_{j}^{T}log\left(q_{j}^{T}\right)\\ p_{i}^{T}=\frac{\exp\left(I_{T}/T\right)}{\sum_{k}^{N}\exp\left(I_{k}/T\right)}\\ q_{i}^{T}=\frac{\exp\left(I_{S}/T\right)}{\sum_{k}^{N}\exp\left(I_{k}/T\right)} \end{array} \end{array}$$

and $p_{i}^{T}$ is the value of the *softmax* output of the teacher model in class I at coefficient *T*; $q_{i}^{T}$ is the value of the *softmax* output of the student model in class *i* at coefficient *T*; in *L*_*hard*_, $q_{i}^{1}$ is the value of the *softmax* output of the student model in class *i* at *T* = 1, *c*_*j*_ is the ground truth value on class *i*, positive labels are taken as 1, and negative labels are taken as 0. The above KD theory is also implemented in this article on YOLO V5-CAcT.

(10) $$\begin{array}{r} \{\begin{array}{c} L_{hard}=-\sum_{j}^{N}c_{j}\log\left(q_{j}^{1}\right)\\ q_{i}^{1}=\frac{\exp\left(I_{S}\right)}{\sum_{j}^{N}\exp\left(I_{j}\right)} \end{array} \end{array}$$

#### Quantitative Storage

In deep learning, model quantization means using fewer bits to store tensors initially stored in floating-point numbers and using fewer bits to perform initial computations in floating-point numbers, but this relies on specific hardware unit support.

Deep learning model parameters are commonly stored using Floating-Point Of 32-bit (FP32); usually, this article can use 16-bit, 8-bit, or even 4-bit to store the model to reduce its storage size. A prevalent practice is to use Integer Of 8-bit (INT8) to store each network parameter for each tensor of each channel in each layer of the model and export the INT8 model to ONNX format for storage after completion. In the model inference phase, this article restores the network parameters to FP32. The weights, intermediate tensor values, and activation values of the model during the operation and the model parameters will be reduced by a factor of 4 due to the substitution of INT8 for FP32 as the model parameter type. Most processors excel at processing INT-type data for embedded platforms, with fewer memory accesses and faster INT8 calculations, generally running 2–4 times faster. Unlike model quantization, the exported INT8 model storage in ONNX format does not rely on any dedicated hardware but only on the support of the inference framework and is therefore widely used in practical production. Quantization storage can be summarized in Equation (11), where *q* is the quantized value of a real number r of type FP32 (Jacob et al., 2018), while the scaling factor *Scale* and *Zp* determine the quantization *q*;

(11) $$\begin{array}{r} r=Scale\left(q-Zp\right) \end{array}$$

Where $x_{float32}^{\max}$ and $x_{float32}^{\min}$ are the maximum and minimum values in the model weight tensor (FP32), respectively, the [*INT*8_min_, *INT*8_max_] are the range of values of INT8. The *float* and *round* functions indicate conversion to single-precision floating-point numbers and rounding. Use the following method to map FP32 to INT8, where *X*_*float*32_ indicates FP32 weights, *X*_*int*8_ indicates INT8 weights due to the storage of the INT8 model of ONNX, quantization of the stored *Scale* and *Zp* values can be saved.

(12) $$\begin{array}{r} Scale=\left(x_{float32}^{\max}-\;x_{float32}^{\min}\right)/float\left({INT8}_{\max}\;-{INT8}_{\min}\right)\; \end{array}$$

(13) $$\begin{array}{r} Zp={INT8}_{\min}-round\left(x_{float32}^{\min}/Scale\right) \end{array}$$

(14) $$\begin{array}{r} X_{int8}=\frac{X_{float32}}{Scale}+Zp,X_{float32}\epsilon \left[x_{float32}^{\min},x_{float32}^{\max}\right] \end{array}$$

Thus, regardless of the framework into which it is loaded, the network parameters can be reduced to FP32 type during the inference phase using the following equation, with the inference time and model accuracy remaining unchanged.

(15) $$\begin{array}{r} X_{float32}=\left(X_{int8}-Zp\right)\;\times Scale \end{array}$$

#### Activate Compression

Deep learning models to fit more features usually require more model parameters, and the industry has generally recognized that multi-parameter models have better performance. In addition, the size of the batch size and the input size of the image also affect the number of model parameters, and a larger batch size affects not only the computational cost but also the training performance (Takase, 2021); in addition, when training a model, in addition to storing model parameters, intermediate activation results and optimizer states are also stored, which requires more memory. It becomes challenging to train these large-scale models with limited GPU memory. Therefore, Chen et al. (2021) proposed the random quantization activation ActNN, which extends the reduced numerical accuracy Activation Compressed Training (ACT) quantization activation proposed by BLPA (Chakrabarti and Moseley, 2019) with the use of a non-uniform quantization strategy proposed by Tiny Script (Fu et al., 2020). ActNN is an excellent algorithm that can quickly compress model parameters without degrading prediction accuracy and supports the commonly used CNN backbone structure, implementing a randomized quantized network layer for most of the commonly used PyTorch nn.Module (Facebook, 2017), ActNN can be used for classification, detection, and segmentation tasks.

Briefly, ActNN implements a dynamic stochastic quantization activation neural network approach that reduces numerical precision by focusing on the activation quantization context, thus enabling quantization compression of weights, activations, and optimizers during training. The quantization process can make the gradient variance affect the convergence. ActNN contains a hybrid accuracy quantization strategy of group quantization and fine-grained quantization, which can approximately minimize the gradient variance during the training process to minimize the gradient variance and achieve a slight loss of accuracy in the 2-bit case. Equation (16), for each training iteration of the l-layer neural network, the forward propagation **F**^(*l*)^ contains the N-feature mapping **H**^(*l*−1)^ with the model parameters Θ^(*l*)^.

(16) $$\begin{array}{r} {\text{H}}^{\left(l\right)}={\text{F}}^{\left(l\right)}\left({\text{H}}^{\left(l-1\right)};\Theta^{\left(l\right)}\right) \end{array}$$

Backpropagation of **G**^(*l*)^ to **H**^(*l*)^ of layer *l* to find the gradient and carry the context **C**( ) to obtain $\nabla_{\Theta^{\left(l\right)}}$, $\nabla_{{\text{H}}^{\left(l-1\right)}}$computed gradient and update the parameters with SGD, calling this robust method precision (FP32) training as follows:

(17) $$\begin{array}{r} \nabla_{{\text{H}}^{\left(l-1\right)}},\nabla_{\Theta^{\left(l\right)}}={\text{G}}^{\left(l\right)}\left(\nabla_{{\text{H}}^{\left(l\right)}},\text{C}\left({\text{H}}^{\left(l-1\right)},\Theta^{\left(l\right)}\right)\right) \end{array}$$

(18) $$\begin{array}{r} \{\begin{array}{c} \nabla_{{\text{H}}^{\left(l-1\right)}}=\nabla_{{\text{H}}^{\left(l\right)}}\Theta^{{\left(l\right)}^{⊤}},\nabla_{\Theta^{\left(l\right)}}={\text{H}}^{{\left(l-1\right)}^{⊤}}\nabla_{{\text{H}}^{\left(l\right)}}\\ \text{C}\left({\text{H}}^{\left(l-1\right)},\Theta^{\left(l\right)}\right)=\left({\text{H}}^{\left(l-1\right)},\Theta^{\left(l\right)}\right) \end{array} \end{array}$$

ActNN to achieve 2-bit activation compression, the contexts **C**( ), Θ^(*l*)^, and $\nabla_{{\text{H}}^{\left(l-1\right)}}$ represented in Equation (17) are each used in a randomized quantization strategy. The computed lossy gradient is an unbiased estimate of the original gradient, as shown in Equation (19) below, i.e., ${\hat{\nabla }}_{{\text{H}}^{\left(L\right)}}=\;\nabla_{{\text{H}}^{\left(L\right)}}$:

(19) $$\begin{array}{r} \begin{array}{c} {\hat{\nabla }}_{{\text{H}}^{\left(l-1\right)}},{\hat{\nabla }}_{\Theta^{\left(l\right)}}={\text{G}}^{\left(l\right)}\left({\hat{\nabla }}_{{\text{H}}^{\left(l\right)}},\overset{ˆ}{\text{C}}\left({\text{H}}^{\left(l-1\right)},\Theta^{\left(l\right)}\right)\right)\\ {\hat{\nabla }}_{{\text{H}}^{\left(L\right)}}=\nabla_{{\text{H}}^{\left(L\right)}} \end{array} \end{array}$$

**Figure 5:**
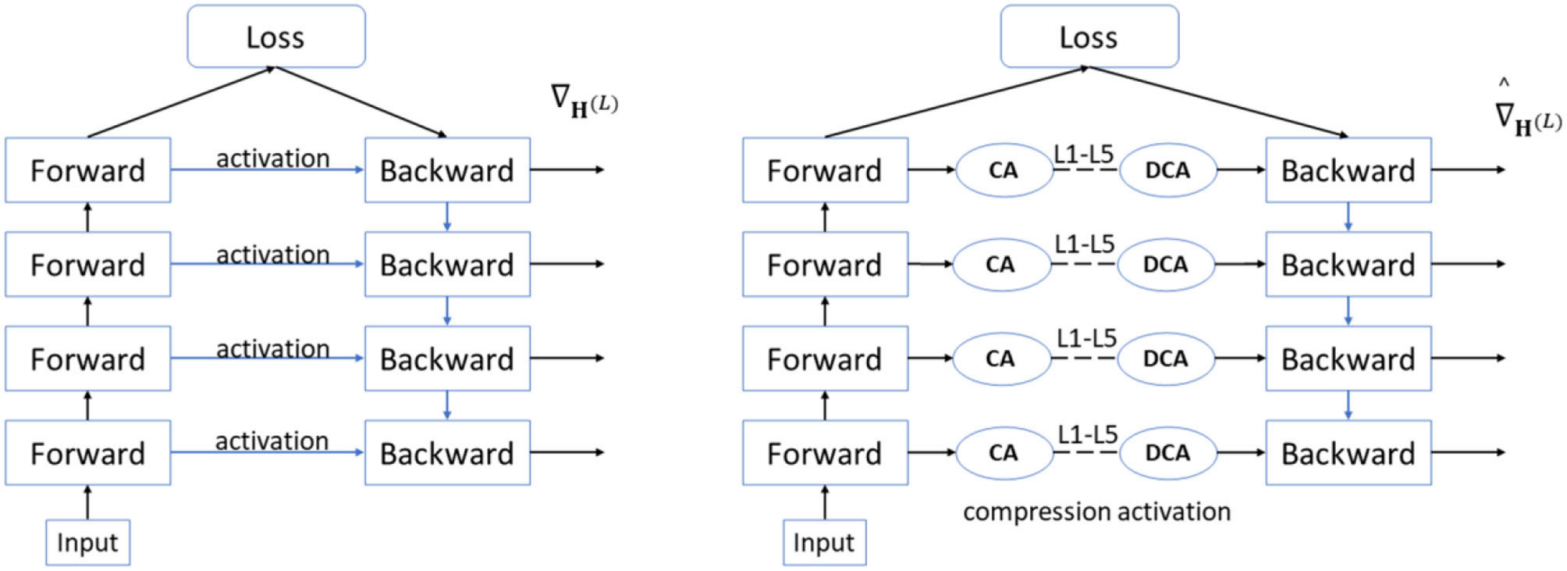
ActNN compression and decompression process.

ActNN dynamically adjusts the hybrid precision quantization strategy at runtime to make better use of the hardware features. Depending on the heterogeneous characteristics between different layers, the compression algorithm keeps more bits for the more essential activation results. In contrast, those activation results that have little impact on the model precision are processed using a compression algorithm above the limit level, assigning an average of 2-bits per activation result, maintaining precision while allowing the model activation parameters can be further reduced. Figure 5 shows that ActNN defines optional compression parameters with increasing compression levels from L1 to L5, where L1 and L2 are compressed using 4-bit per-group quantization, but L1 allows 32-bit quantization and processes only the convolutional layers; L3–L5 are compressed at 2-bit using fine-grained- mixed-precision, swapping, and defragmentation compression at 2-bit, respectively, which act on the activation results of all layers, and the specific processing effect depends on the proportion of the original model you process using the ActNN module. The processing is only done in training, and the detection process is not involved. In addition, as shown in Equation (20), the compression algorithm used in L1–L5 is a superposition of the previous compression level. In the training process, under the same hardware conditions, the higher the compression level, the longer the decompression time of the activation results during backpropagation, and the slower the training speed, from the perspective of adjusting the parameters and data, increasing the batch size and using high-resolution images will increase the Compression Activation (CA) and Decompression Activation (DCA) time, slowing down the model convergence efficiency.

(20) $$\begin{array}{r} L1⊊L2⊊L3⊊L4⊊L5 \end{array}$$

#### YOLO V5 With ActNN Integration

**Figure 6:**
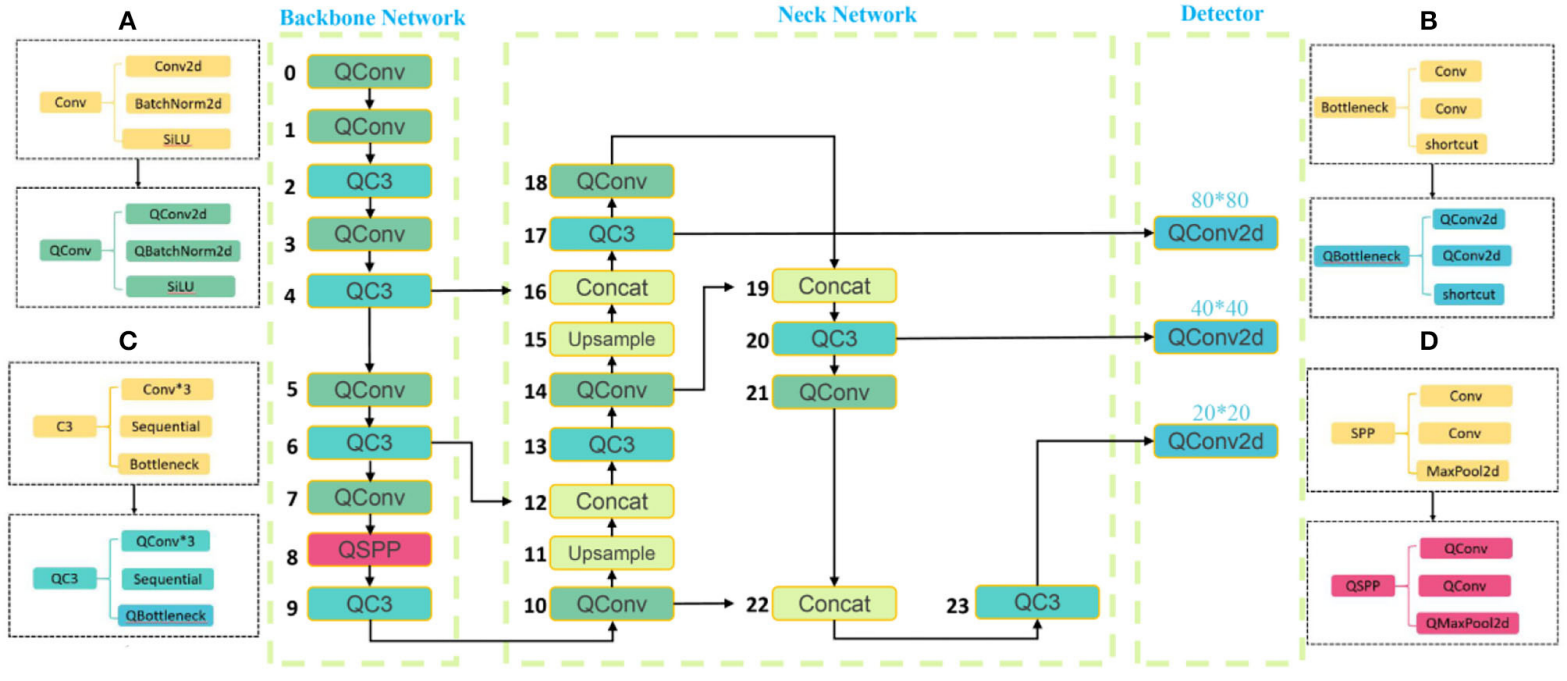
Integration of YOLO V5 model structure using ActNN.

The YOLO V5 network is based on the PyTorch implementation, and the three main structures in the model contain modules that are directly or indirectly inherited from the module under the nn package. These modules perform the primary operations from feature extraction, and feature fusion, to classification output, and are composed of convolutional computation, pooling operation, BN (Batch Normalization), and activation function as essential components to measure the size of the number of module parameters. Compressing the corresponding fine-grained parameters of the constituent modules enables effective optimization from the model base structure without changing the overall functional structure of the model and thus also without affecting the model's performance. ActNN implements most of the modules with compressed parameters, where QConv and QConvTranspose have versions of convolutional kernel modules corresponding to three different sizes, and QBatchNorm performs BN operations on three different versions of modules, in addition to the commonly used ReLU, Dropout, and MaxPool2d operations. In this study, the original network structure of YOLO V5 shown in Figure 2 is improved so that YOLOV5 integrates AcTNN, RA, FocalLoss, SmoothBCE, and Early Stopping, and YOLO V5-CAcT is proposed, which inherits all the features of YOLO V5 and adds the functional properties of activation compression parameters according to the technical route. For the four main model structures of YOLO V5, the corresponding implementations in this study are named YOLO V5s-CAcT, YOLO V5m-CAcT, and YOLO V5l-CAcT. Briefly, this article adopts AcTNN to integrate and replace some modules of the original network structure, and QConv, QBottleneck, QC3, and QSPP are designed to replace the corresponding modules, as shown in Figures 6a–d, and Upsample and Concat are still preserved because they are only involved in parameter passing and do not bring additional computational overhead. Finally, QConv2d is replaced with Conv2d in the Detector structure to obtain the model's overall structure after integration in Figure 6.

#### Model Conversion

The fast crop leaf disease detection experiments are implemented based on the PyTorch framework, a deep learning framework developed by Facebook that is widely used in the industry for its efficient computational performance and good usability. However, PyTorch model files are not directly usable by other inference frameworks. The most common way to deploy PyTorch models is to convert them into an open format and then use other inference frameworks to convert the open format to their own. ONNX (Microsoft Facebook, 2018) is a generally accepted open format as a standard format for AI models, allowing engineers to move deep learning models between different frameworks. In addition, the use of ONNX will significantly reduce the probability of accuracy degradation after model transformation.

#### Simplify Operator and Model Deployment

Different deep learning frameworks generally implement different operators to perform the same operation. ONNX, the model standard, implements most operators, but when other models are converted to ONNX, a simple operation of other models will become redundant and complex in ONNX. Operator fusion combines multiple adjacent operators in ONNX into a linear block operator without storing redundant intermediate results in memory, reducing the number of accesses and therefore significantly reducing execution time, especially in GPUs and NPUs.

**Figure 7:**
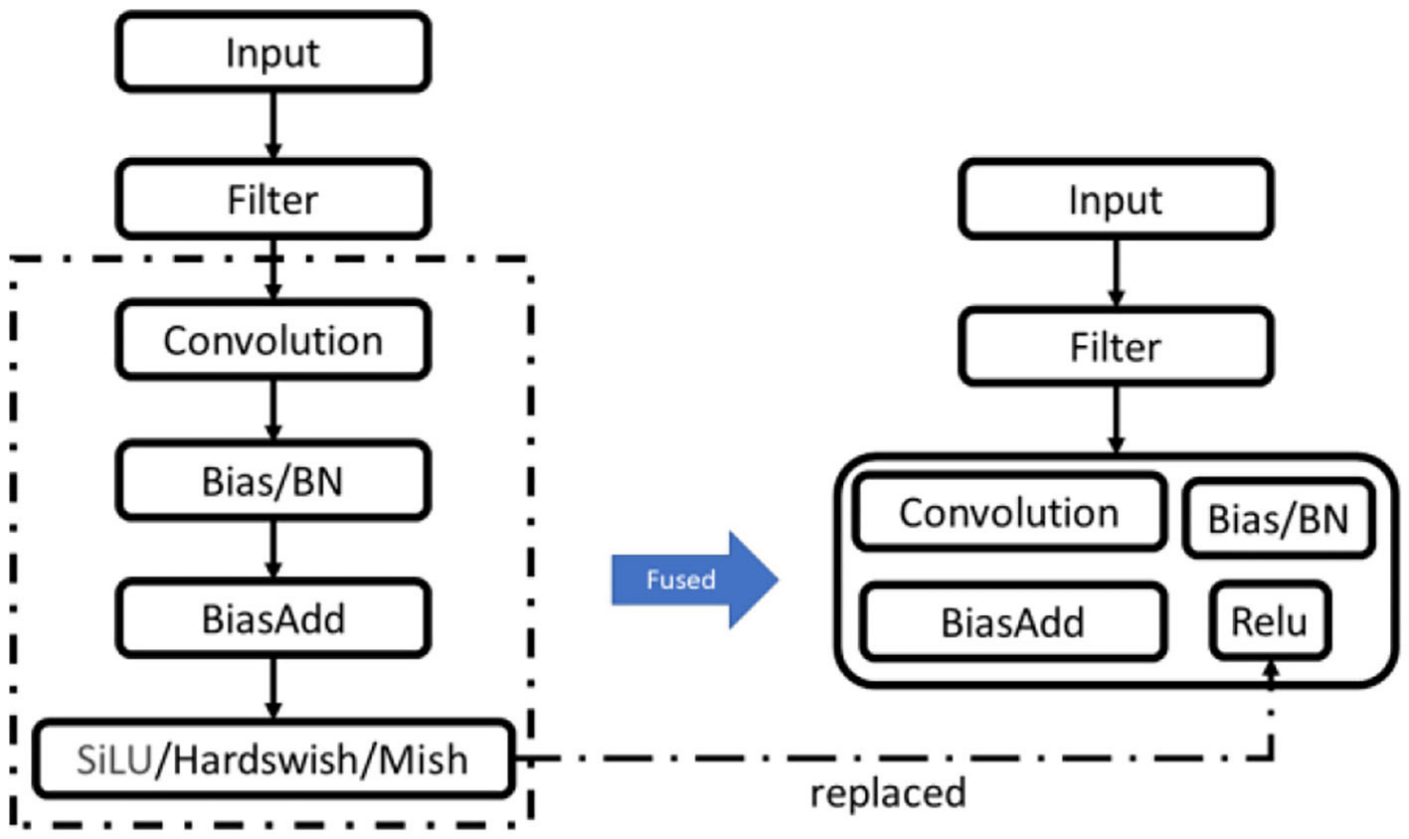
layer fusion and data reuse.

Currently, the convolutional layer context layers that generate the model after training are optimizable. Most of the inference framework operations in the inference phase can be reduced to linear operations, and simplifying the model structure generally requires linear optimization using fusion techniques (Chitty-Venkata and Somani, 2020). The sequence of steps involved in a single convolutional layer are convolutional operations, bias addition, batch-normalization-operators (BNO), and activation functions (SiLU, Hardswish, and Mish); the fusion mechanism combines these steps to form a single step, i.e., they are executed simultaneously, as shown in Figure 7.

To effectively match the deployment applications of real agricultural scenarios, further achieve model acceleration, and reduce the hardware burden, this article converts the simplified model into an NCNN model. It then loads it through the NCNN C++ API. The choice of using Tencent's Neural Network Inference Framework (NCNN) (Tencent, 2022) is because it is a high-performance neural network inference computing framework optimized for ARM mobile platforms, which is implemented entirely in C++ and does not rely on any third-party libraries. It can be quickly and efficiently deployed on multiple device terminals.

### Dataset

#### PFD Dataset

The PFD dataset is based on the AI Challenger (Zhang et al., 2020) and PLD (Potato Disease Leaf) open-source datasets (The PLD dataset is available at https://www.kaggle.com/datasets/rizwan123456789/potato-disease-leaf-datasetpld) and some of the PlantVillage crop disease data (The PlantVillage dataset is available at https://www.kaggle.com/datasets/soumiknafiul/plantvillage-dataset-labeled). Almost all researchers in crop disease identification have used the PlantVillage dataset in their studies. The Plant Village dataset contains 31,397 healthy and diseased leaf images, which consists of 256 × 256 size JPG color images divided into 25 categories (20 diseased images, 5 healthy images, and 5 crop species) by species and disease. The PLD dataset is a collection of 4,072 potato disease images from the central region of the Punjab province of Pakistan, and the diseases include Early Blight, Late Blight, and Healthy. The AI Challenger dataset was divided into 61 categories by species, disease, and degree, with 10 species and 27 diseases (24 diseases had both general and severe degrees), but there were categories with imbalances or tiny sample sizes. The selection of AI Challenger as the research dataset can cover more crop diseases and better reflect the performance advantages of the model.

#### Building the Dataset

The PFD dataset contains 52,589 crop disease image data with an image size width of 256 and height between 256 and 512, mainly composed of the AI Challenger dataset and part of PlantVillage and PLD. The analysis by plant pathologists revealed six categories of crop diseases with unbalanced or incorrect categories in the AI Challenger dataset. Tomato Bacterial Spot Bacteria general, Tomato Bacterial Spot Bacteria serious, Tomato Target Spot Bacteria general, and Tomato Target Spot Bacteria serious 4 categories have serious labeling errors. This article extracted and replaced the Tomato Bacterial Spot Bacteria and Tomato Target Spot Bacteria in the PlantVillage dataset Color Images to reduce the original four categories of diseases to two categories. The remaining two categories of unbalanced samples are shown in Tables 1, 2 summarizes the three datasets. Using the image data generator method of Python's Albumentations library, 5 data enhancement techniques were applied to 2 types of unbalanced diseases present in the dataset to overcome overfitting and enhance the diversity of the dataset.

1. Spin: Rotating the images randomly by 0°, 90°, 180°, and 270°, simulating the randomness of shooting angles under natural conditions, will not change the relative positions of diseased and healthy crop features.
2. Color jitter: Identify crop disease scenes mainly in the field, which are affected by weather, and change the brightness, contrast, and saturation of images with 0.2 random probability to simulate the differences of images taken in different weather photos.
3. Blur: Motion blur or median filtering is added randomly to the images to simulate different defined images taken in a field environment with a random probability value of 0.2.
4. Noise: Add gaussian noise to an image with Multiplicative noise is used to generalize multiple images and shield the differences of many factors, such as image acquisition equipment and the natural environment.
5. Resize: After the above steps, the image's resolution is extended or scaled to 512 × 512 pixels by filling 0 pixels.

**Table 1:** Weak dataset enhancement.

**Type**	**Origin**	**Augmentation**
	**images**	**images**
Cedar apple rust serious	46	230
Grape leaf blight fungus general	70	350

**Table 2:** AI challenger, PLD, and PlantVillage dataset summary.

**Dataset**	**Training dataset**	**Validation dataset**	**Testinging dataset A**	**Testinging dataset B**	**Error labels**
AI challenger	31,718	4,540	4,514	4,513	76
PLD	3,251	416	405	/	/
PlantVillage	Tomato bacterial spot bacteria	Tomato target spot bacteria			
	1,322	1,402			

**Figure 8:**
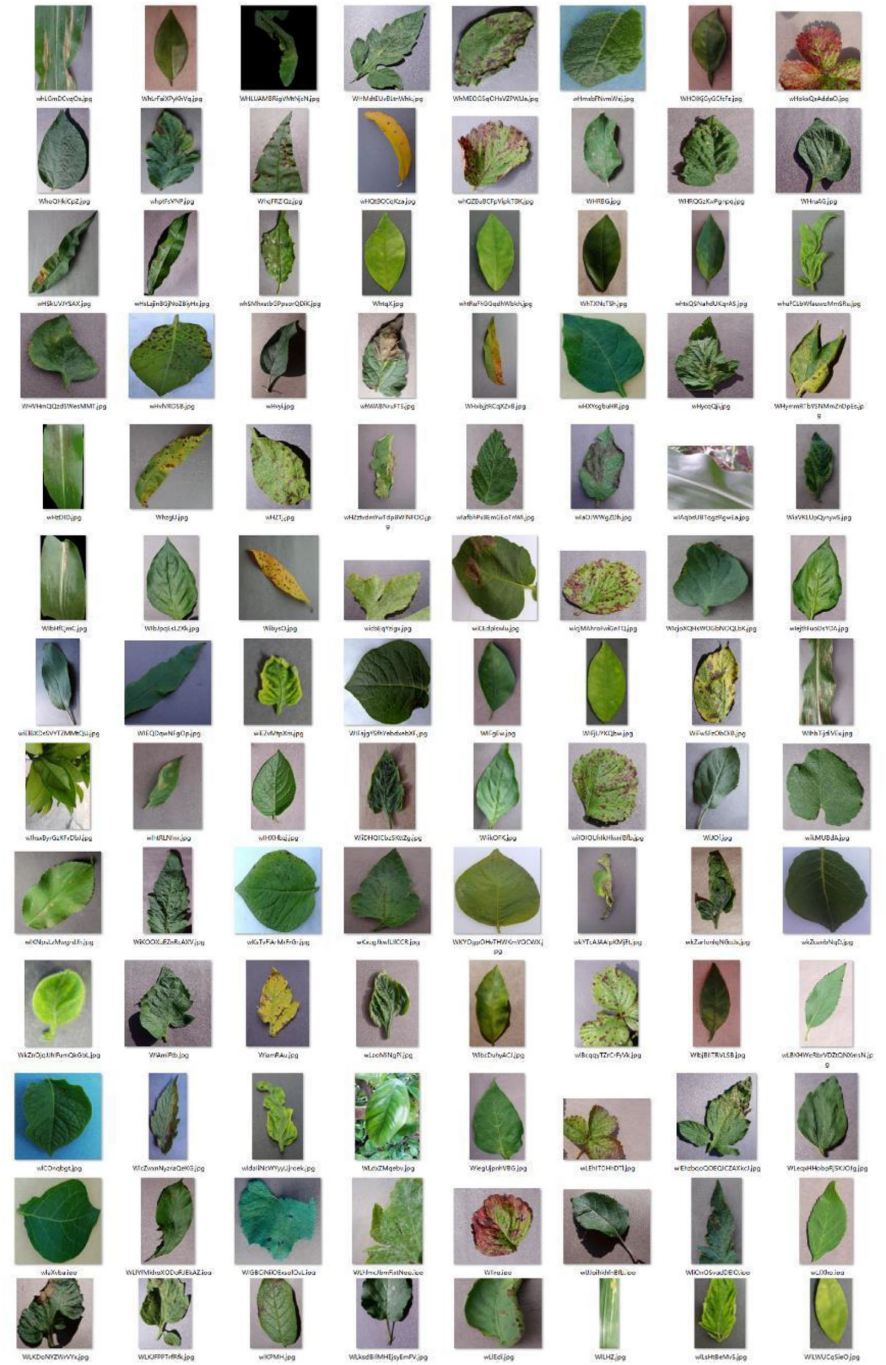
Sample images of the PFD dataset.

By the above data enhancement method, the sample size of each category was expanded by five times, and the enhanced dataset of these four crop disease categories contained 580 images. Later, after data analysis, it was found that the data set had less data on potato leaf disease-related species, and due to the rapid development of the potato seed industry, 4072 images of data from the PLD data set were selected to make up for this discrepancy. Finally, Convert the PFD dataset to VOC format, after statistics, the PFD dataset label categories consisted of crop species, disease name, and disease degree, including 59 disease categories, 10 crop species, and 27 disease classifications (of which 22 diseases have two degrees of classification: general and severe), and 10 healthy crop classifications, Figure 8 shows the sample images of the PFD dataset.

#### Data Pre-processing

In deep learning, to obtain better consistent classification results and better feature extraction, it is usually necessary to pre-process the dataset. there are more human-labeled data in the PFD dataset samples, which may have duplicate samples, thus causing the final result of the model solution to be biased toward reducing the training error of this part of the samples at the expense of the training error of other samples, i.e., OverSampling. In this article, we measure image similarity in four aspects: hue, saturation, brightness, and structure of the image, and use the Structural Similarity Index (SSIM) algorithm (Bakurov et al., 2022) with a similarity threshold of 0.95 (maximum value of 1) to filter out similar images, and considering that similar image samples affect the generalizability of the model, for each category of images below the similarity threshold are retained. The final de-weighted dataset was 51,772, and the training validation set and test set were divided 9:1 using the Hold-Out method. In contrast, the training and validation sets continued to be divided 8:2, as shown in Table 3.

**Table 3:** Dataset partition composition.

**Dataset**	**Samples**
Training set	37,239
Validation set	9,310
Testing set	5,172

#### Training Data Augmentation

In deep learning, different data enhancement techniques are applied to the training, to overcome the spillover, while the generalization capability and robustness of the model can be improved (Sambasivam and Opiyo, 2021). Therefore, in this article, an adaptive image enhancement technique is used to employ the Repeated Augmentation (RA) sampling strategy (Fort et al., 2021), where each mini-batch drawn from the training set corresponds to a different image-enhanced version of the same sample combination, and the sample combinations of the mini-batch are guaranteed to be non-completely independent, an approach that allows the model to more easily learn the enhanced invariant features. The above strategy is combined with YOLO V5-CAcT in the PyTorch framework to enhance the image data and select important hyperparameters to be used in subsequent model training. For this purpose, the YOLO V5s-CAcT model is selected in this article. The RA strategy is used, the training images are rescaled to 384 × 384 sizes before being input to the network, 300 cycles are iterated under hyperparameter evolution, and the test fluctuation range of the lesser image enhancement hyperparameters are selected as the final parameters for the subsequent training as shown in Table 4.

**Table 4:** Image augmentation parameter setting.

**Params**	**Values**
hsv_h	0.01430
hsv_s	0.63383
hsv_v	0.34523
translate	0.09936
flipud	0.01850
fliplr	0.50000
mosaic	0.91901

## Experiment

In this section, the experimental platform for this article is documented and the model training parameters used are summarized. Rigorous experiments were conducted on the techniques mentioned in the technical routes, and the conclusions demonstrate the feasibility of the solutions in this article. The two technical routes successfully train, optimize and deploy deep learning models with significant inference speed and very high accuracy compared to other models.

### Experimental Setup and Training Parameters

#### Evaluation Platforms

The operating platform for this experiment is the Nettrix X640 G30 AI server with Ubuntu 20.04 OS environment, two Intel(R) Xeon(R) Gold 6226R CPUs @ 2.90GHz, two N-VIDIA GeForce RTX 3090 GPUs, 256G RAM, 7.5T solid-state drives. The training environment was created by Anaconda3 and configured with Python 3.9.5, PyTorch 1.10.1, and Torch Vision 0.10.1 artificial neural network libraries. Also, the CUDA 11.1 deep neural network acceleration library was used.

#### Training Setting

In the model used in this study, YOLO V5-CAcT represents the network structure that integrates AcTNN, model pruning, and knowledge distillation according to technical routes 1 and 2, while YOLO V5 represents the original network structure. The loss function uses BCELoss (Xu et al., 2022), the optimizer uses SGD, the batch size is 128, the input image size is 384, the learning rate is initialized from 0.0032 and finally to 0.12, the momentum parameter is 0.843, the weight decay is set to 0.00036, and the preheating parameter 5 is used to ensure that the model has some prior knowledge of the data. Other parameters were kept as default, and the model with the highest accuracy was selected as the pre-trained model by pre-training with 1,000 epochs on the PFD dataset and fine-tuning the model several times. Although this article uses Table 3 parameters for image broadening, likely, there is still a problem of imbalance between positive and negative samples in the sample, so the original loss function is changed using FocalLoss (Yun et al., 2019) and SmoothBCE (Zhang et al., 2019), and the Flgamma is set to 1.5, SmoothBCE serves to reduce the possibility of model overfitting, and the batch size is changed to 64, the input image size is 512, and other hyperparameters are set the same as pre-training. The convergence rate of model training is related to the specific dataset; when it appears that the model performance keeps growing in <0.01 steps, the training will not stop, and the model does not converge well; at this time, the best way is to monitor this problem and intervene in time, the Early Stopping (Dodge et al., 2020) early stopping mechanism appears to be an excellent solution to this problem. This article integrates the early stopping mechanism with YOLO V5-CAcT, and the parameter is set to 100. In addition, in this article, the model is trained by fine-tuning five times, executing 300 epochs each time, recording the results with the highest precision, and then using the best results as the input for the next step.

### Results

#### Evaluation of Model Training

In this subsection, several YOLO V5 networks with different parametric quantities will be used for comparison: YOLO V5s, YOLO V5m, YOLO V5n(Nano), and trained according to the training setup in section Experimental Setup and Training Parameters, i.e., using the training method of the initial model with the improved policy model. To assess performance, average accuracy evaluation metrics recognized in the field of image detection are used to evaluate detection results, including precision (*DP*), recall (*DRR*), F1-score (F1), and average accuracy (*mAp*@0.5).

(21) $$\begin{array}{r} DP=\frac{T_{P}}{T_{P}+F_{P}} \end{array}$$

(22) $$\begin{array}{r} DRR=\frac{T_{P}}{T_{P}+F_{N}} \end{array}$$

(23) $$\begin{array}{r} F1=2\times \frac{DP\times DRR}{DP+DRR} \end{array}$$

(24) $$\begin{array}{l} AP@0.5=\frac{1}{n}\overset{n}{\underset{i=1}{\sum }}DP_{i}=\frac{1}{n}DP_{1}+\frac{1}{n}DP_{2}+\ldots \\ \;+\frac{1}{n}DP_{n} \end{array}$$

(25) $$\begin{array}{r} m\left(Ap\right)@0.5=\frac{\sum_{i=1}^{Q}{AP@0.5}_{i}}{Q} \end{array}$$

Where *T*_*P*_, *F*_*P*_ and *F*_*N*_ in Equations (21) and (22) referred to the number of correct checkboxes, incorrect checkboxes, and missed checkboxes, respectively. *F*1 of Equation (23) is a comprehensive measure of the accuracy and completeness of the search. The calculation of *m*(*Ap*)@0.5 depends on *AP@*0.5, where *AP@*0.5 is defined as when the IOU threshold is taken as 0.5; for a specific category of samples with N correct checkboxes, each additional correct check box will correspond to a *DP* value, and the average of N DPs is obtained for the category *AP@*0.5, which is calculated in Equation (24). *m*(*Ap*)@0.5 is defined as the mean value of *AP@*0.5 under all categories, as shown in Equation (25), Q refers to the total number of detected categories, and there are 59 crop disease categories in this article, so it is 59 here. *m*(*Ap*)@0.5 as the mean cumulative value of the multi-category detection rate can show the comprehensive performance of the multi-category model as a whole, and it can be defined as an essential index to measure the comprehensive performance of the model. The difference between *F*1 and *m*(*Ap*)@0.5 is that *m*(*Ap*)@0.5 reflects the high accuracy rate and the high recall rate.

In addition to this, to further compare the methods proposed in this article to improve accuracy, we used ablation experiments to approach the detection task, all three methods are based on YOLO V5s-CAcT, including the following:

1. YOLO V5s-CAcT1: Data Augmentation method based on the RA sampling strategy is used.
2. YOLO V5s-CAcT2: Modify the original loss function, add FocalLoss and SmoothBCE loss function to the original loss function.
3. YOLO V5s-CAcT3: Simultaneous use of Data Augmentation based on RA sampling strategy and use of FocalLoss with SmoothBCE loss function.

In this article, experiments were conducted on the AI Challenger and PFD datasets, and the experimental results for these two datasets are shown in Table 5. Table 5 shows the accuracy performance metrics when comparing using the three-class approach of this article and the three-class model of YOLO V5, which are generated from the latest research methods.

**Table 5:** AI challenger and PFD dataset model training results.

**Model**	**AI challenger**				**Plant fruit disease (PFD)**			
	**DP(%)**	**DRR(%)**	***F*1 (%)**	**mAp@0.5(%)**	**DP(%)**	**DRR(%)**	***F*1(%)**	**mAp@0.5(%)**
YOLOV5n(Nano)	74.2	85.4	79.4	82.7	75.8	87.7	81.7	84.4
YOLOV5s	79.5	87.9	83.5	88.7	87.2	92.6	89.8	94.3
YOLOV5m	82.9	89.8	86.2	91.8	88.7	93.9	91.2	96.5
YOLO V5s-CAcT1	79.2	88.5	83.5	89.2	86.5	92.9	89.5	93.7
YOLO V5s-CAcT2	80.6	87.5	83.9	89.1	91.2	90.2	90.6	95.8
YOLO V5s-CAcT3	81.2	88.7	84.7	90.1	90.7	92.3	91.5	95.6
**Dataset**		**YOLOV5n(Nano)**		**YOLOV5s**	**YOLOV5m**		**YOLO V5s-CAcT3**	
		**mAp@0.5(%)**						
PFD includes PLD		84.4		94.3	96.5		95.5	
PFD without PLD		83.9		94.1	96.4		95.3	

Among these six models, the results of the PFD dataset are better than those of the AI Challenger dataset for all models of the same level. Regarding accuracy, the three methods based on the original YOLO V5 model, without considering the methods proposed in this article, have at least 1.6, 2.3, and 1.7% advantages in *DP*, *F*1, and *mAp@*0.5, respectively. In addition, during the training process, the four unbalanced crop disease categories of AI Challenger had different degrees of impact on the overall performance of the original YOLO V5 model. This phenomenon can be attributed to the severe shortage of sample size, especially the presence of mislabeled samples among them, making the accuracy rate worse. The PFD dataset removed the two crop disease categories that were mislabeled. The data images were regenerated using data enhancement for the other two categories with fewer samples, both of which showed better performance metrics than the original dataset in terms of experimental results; The post-supplemented potato leaf disease data also did not affect the model's overall performance, and the individual performance metrics were higher than those of the PFD dataset without the addition of PLD. Therefore, the method of constructing the PFD dataset proved to be successful.

Among the three methods proposed in this article, YOLO V5s-CAcT3 has better all-around performance than the other two methods. It outperforms the original YOLO V5s model in the AI Challenger and PFD datasets. The lowest performance metric selected from the three methods was compared to the original YOLO V5s model, with *mAp@*0.5 0.4% higher in AI Challenger and 1.5% higher in the PFD dataset. In addition, an interesting phenomenon is that the *mAp@*0.5 of YOLO V5s-CAcT1 in AI Challenger is better than that of the original YOLOV5s model. At the same time, in the PFD dataset, it is lower than that of the original YOLO V5s model by 0.6%. After analysis, the main reason for this phenomenon is that the pre-trained hyperparameters obtained using the RA sampling strategy in this article are built on top of the AI Challenger. A negative gain in performance occurs by applying it to the PFD; In YOLO V5s-CAcT2, a new strategy is used to recover and improve performance by 1.5%, so it is clear that YOLO V5s-CAcT1 and YOLO V5s-CAcT2 have some complementary effects. In addition, YOLO V5s-CAcT3 has a somewhat more significant improvement on DRR, with 1.2 and 2.1% improvement for the two datasets, respectively. In summary, the two strategies proposed in this article successfully improve the original YOLO V5s model.

To compare the proposed YOLO V5s-CAcT3 with other advanced methods, six well-known CNNs, such as Faster RCNN, SSD, YOLO v3, YOLO V4, and YOLO V5s, were selected as baseline methods for comparison experiments. By applying transfer learning methods, pre-trained weights are obtained on ImageNet (Gu et al., 2021) to initialize the weight parameters, and Softmax is embedded into the network for classification. The hyperparameters assigned to the network are a learning rate of 0.001, a momentum of 0.9, a batch size of 64, and a stochastic gradient descent (SGD) solver with unrestricted epochs for each model and multiple fine-tuning to ensure optimal convergence.

As shown in Table 6, the proposed method obtains competitive performance and provides better results than other comparative methods. The proposed method achieves an average accuracy of 95.6%, which exceeds that of YOLO V4, one of the most advanced models available, by 3.2%, and is the best of all algorithms. Comparing the size of all models, this model is relatively the smallest and has the highest accuracy. Further, the PFD training set gets pre-trained models with image enhancement hyperparameters, making it easier for the network to learn the features of plant disease images and obtain optimal weight parameters, thus improving the accuracy of crop disease identification. In contrast, the other methods are single neural networks that do not achieve optimal results despite applying transfer learning and fine-tuning.

**Table 6:** Test results of different models in the PFD dataset.

**Models**	**DP(%)**	**DRR(%)**	***F*1(%)**	**mAp@0.5(%)**
Faster RCNN	86.7	89.1	87.8	89.9
SSD	84.6	87.9	86.2	87.1
YOLO v3	81.9	86.2	83.9	84.7
YOLO v4	87.5	93.8	90.5	92.4
YOLO V5s	87.2	92.6	89.8	94.3
YOLO V5s-CAcT3	90.7	92.3	91.5	95.6

The results of this study were compared with the results of other studies shown in Table 7. Rashid et al. (2021) and Mathew and Mahesh (2022) used the same dataset as this study. The accuracy of all these studies is lower than the model presented in this article. Even the accuracy of the YOLO V5 model used by Mathew and Mahesh (2022) was 4.8% lower than our study. By comparing the model accuracy of the different number of disease categories, the model accuracy of Zhang et al. (2020), Gao et al. (2021), Sharma et al. (2021), Zhao et al. (2021), and Al-Wesabi et al. (2022) is higher than our results, which is due to the smaller number of disease categories (up to 10 categories). Our study required the identification of up to 59 plant disease categories, which exceeded at least 85% of the disease categories in other studies and reduced the accuracy by up to 4.5% relative to other studies. Overall, the model has excellent overall performance and high diagnostic accuracy for many plant diseases.

**Table 7:** Results in the article compared with other state-of-the-art results.

**Paper**	**Dataset**	**Model**	**Classification**	**Precision (%)**	**Recall (%)**	**F1-score (%)**	**Accuracy (%)**
Zhao et al. (2021)	PlantVillage	ResNet-50+SeNet	10-class	96.77	96.81	96.79	96.81
Mathew and Mahesh (2022)	PlantVillage	YOLO V5	2-class	–	–	–	90.7
Afzaal et al. (2021)	–	EfficientNet	6-class	74.00	74.00	76.00	–
Sharma et al. (2021)	Potato leaf dataset	Proposed CNN	3-class	99.63	99.6	99.69	99.58
Sharma et al. (2021)	Rice leaf disease	Proposed CNN	4-class	98.00	98.00	98.00	97.66
Gao et al. (2021)	–	SegNet	2-class	–	–	–	99.8
Our model	AIChallenger+ Plant Village+PLD	YOLO V5-CAcT	59-class	90.70	92.30	91.50	95.50
Rashid et al. (2021)	PLD	PDDCNN	3-class	–	–	–	86.38
Zhang et al. (2020)	AIChallenger	Faster RCNN-res101	4-class	–	–	–	97.18
Al-Wesabi et al. (2022)	New plant diseases dataset	AIE-ALDC	4-class	99.40	99.40	99.40	99.70

**Figure 9:**
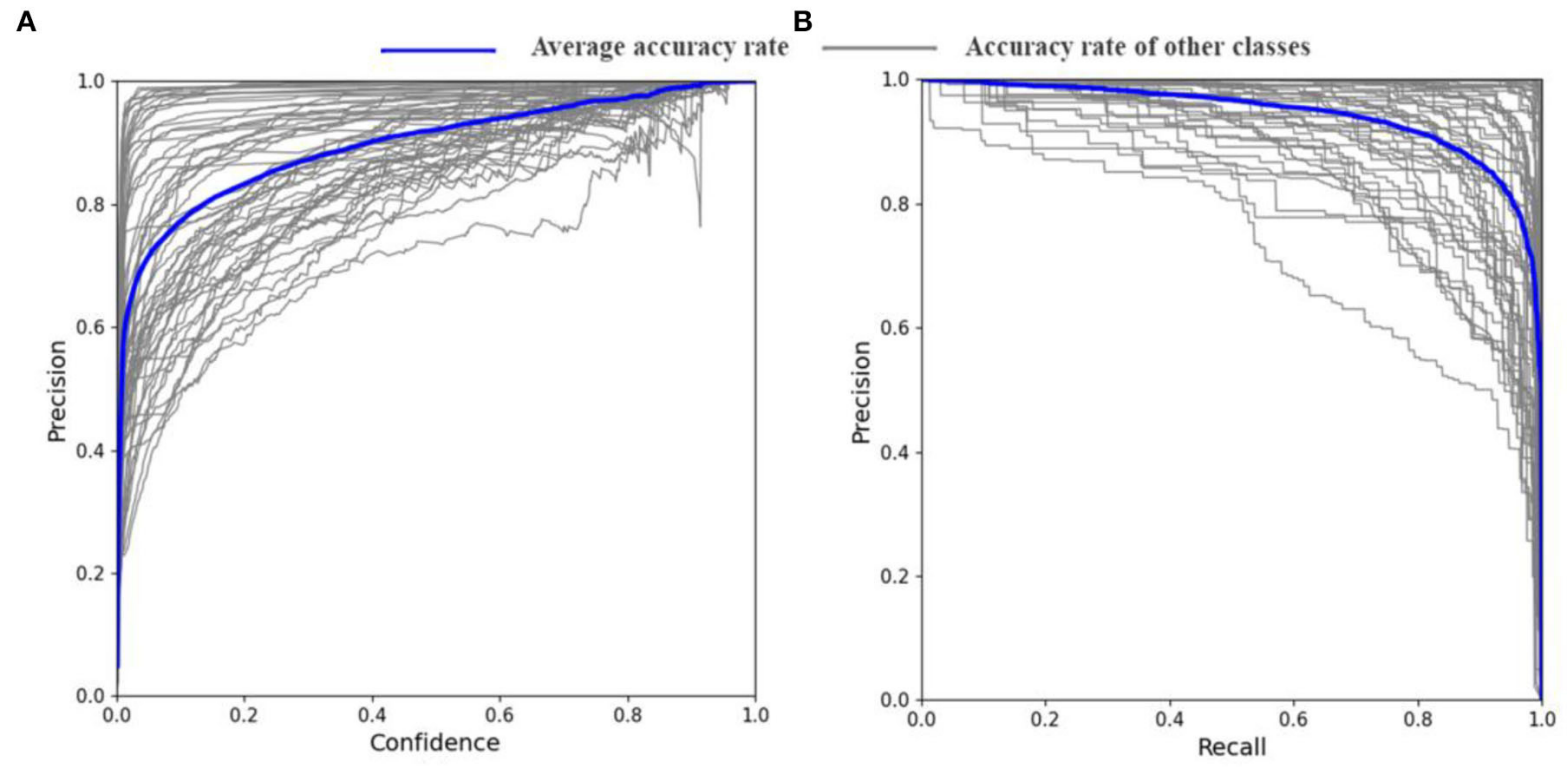
Confidence accuracy curve and recall accuracy curve. **(A)** Precision-confidence curves of the YOLO V5s-CAcT3 model on the PFD dataset. **(B)** PR curves of YOLO V5s-CAcT3 model on the PFD dataset.

The YOLO V5s-CAcT3 is the result of the improvement of both strategies. To further illustrate the performance of the model on the PFD dataset and to demonstrate the crop disease accuracy of each category in the constructed PFD, the performance of the YOLO V5s-CAcT3 model is visually depicted in this article using Figure 9, with the blue line indicating the average accuracy of all crop disease categories and the brown line indicating the accuracy of each crop disease. Figure 9A is a composite indicator of the continuous variables of confidence and accuracy; the more the upper right corner of Figure 9A is closer to the accuracy 1 line, the better the classifier is working; With confidence levels above 0.8, 92% of the crop disease categories maintained an average accuracy above 0.9, whereas in general, only 0.75 was required. Although one of the crop disease categories was less accurate at confidence levels above 0.8, it remained above 0.75 and met the requirements. Figure 9B is a composite indicator of the continuous variation of recall and accuracy. The larger the area of the lower half of the blue curve in Figure 9B indicates that the classifier is working better in the limit, with a recall above 0.9 corresponding to accuracy above 0.85 and have 72% of the crop disease categories meeting this condition. There is also a poorer crop disease category in Figure 9B, with a recall of 0.7 and an accuracy of only around 0.6. This is the same category as the poorer crop disease category in Figure 9A. In summary, the YOLO V5s-CAcT3 model has shown good performance under both limits operating state curve validations.

#### Analysis of Model Compression

##### Model Prune Results

**Figure 10:**
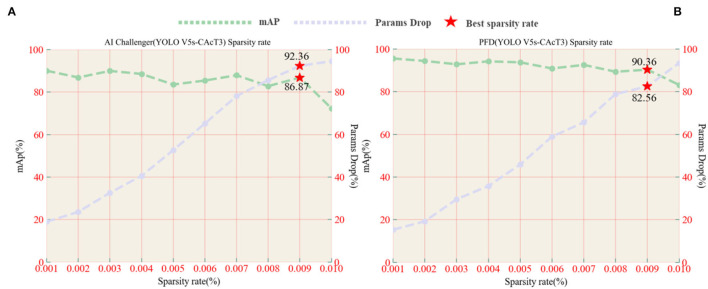
**(A)** AI Challenger accuracy, parameter drop, and sparsity curves vs. **(B)** PFD accuracy, parameter drop, and sparsity curves.

A necessary process prior to pruning is to perform sparse training to find the most appropriate sparse rate, which is an essential parameter for controlling pruning depth. In order to find the optimal sparse rate, model sparse training experiments were conducted on the original model at sparse rates from 0.001 to 0.1 to investigate the effect of sparse rate variation on model accuracy and model parameter degradation. Using YOLO V5s-CAcT3 as the original model, Figure 10 shows the performance of the model, including the average accuracy and rate of parameter decline under the AI Challenger vs. PFD dataset, with the brown line indicating the average accuracy of the model and the light blue line indicating the percentage decline in the model parameters. The horizontal coordinates of the red pentagrams indicate the corresponding optimal sparsity, and the vertical coordinates indicate the average accuracy of the model or the percentage decrease of the model parameters, respectively. The analysis shows that the model's accuracy with sparse training decreases with increasing sparsity, while the rate of parameter decline of the model increases with increasing sparsity. The optimal sparse state of the model is chosen to ensure that the average accuracy of the model is the maximum of the critical state, i.e., the value before the average accuracy drops sharply, and also to satisfy that the rate of decline of the model parameters is as large as possible, in addition, the value of the horizontal coordinate corresponding to the maximum of the critical state is the optimal sparse rate.

Analysis of the experimental results shows that choosing the optimal sparsity rate ensures that the model accuracy is close to that of the original model while also reducing the number of parameters in the model. However, when choosing a sparse rate higher than the optimal sparse rate, although it can further reduce the number of model parameters, it cannot prevent the model accuracy from dropping sharply, as shown in Figure 10B, using a 0.01 sparse rate compared with 0.009 sparse rates will lead to a rapid decrease inaccuracy, so 0.009 is chosen as the optimal sparse rate for the following experiments of the YOLO V5s-CAcT3 model in this article. It is worth noting that the YOLO V5s-CAcT3 model has similar sparsification training curves under the AI Challenger and PFD datasets, and both show a dramatic change in performance after 0.009 sparsity, suggesting that the redundancy parameter threshold in the YOLO V5s-CAcT model space is around 0.009 sparsity, which is a guideline for other applications of sparsity.

**Figure 11:**
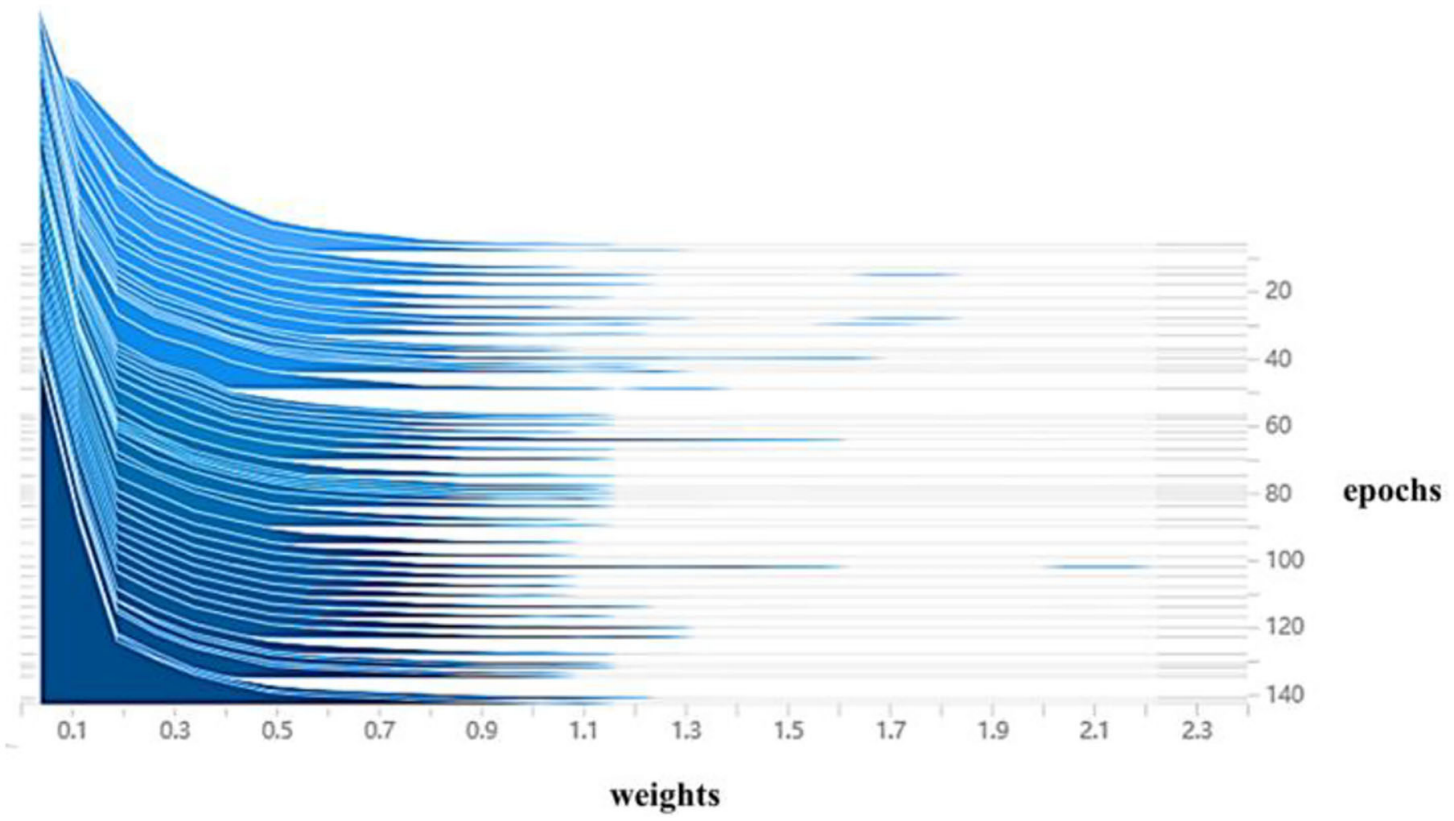
Histogram of model weight change during sparse training.

The BN layer weight histogram is an essential indicator of the sparse training status. In this article, the training parameters were fine-tuned concerning section Experimental Setup and Training Parameters for 200 iterations of the training settings at a sparse rate of 0.009. As shown in Figure 11, the horizontal coordinates in the figure indicate the weighting factors and the vertical coordinates indicate the number of iteration cycles. The blue slice corresponding to the number of iterative cycles is the histogram of weights at a given cycle, viewed from the inside out as a process of superimposing individual histograms, The slices are organized by iteration cycle, with the more advanced slices indicating the newer the current sparse state slice of the model; the weight coefficients in the figure gradually converge to 0 as training progresses, with only some of the weight coefficients not decaying to 0, indicating that the weight coefficients are gradually becoming sparse. Until 140 iterations, the weight coefficients tend to stabilize, indicating that the sparse training has reached a steady state, and the stable sparse state will be used as the basis for subsequent model pruning.

Next, this article uses the sparse model with optimal sparse parameters for pruning. It uses the commonly used Slim-Filter-Pruner (Liu et al., 2017), L1-Norm-Pruner, and L2-Norm-Pruner (Li et al., 2016) pruning algorithms as a reference group to demonstrate the effectiveness of the pruning method in this article. Table 8 shows the effect of different pruning algorithms on the accuracy and parameters of the final pruning model, with the method in this article (FPGM-YOLOV5) significantly outperforming the other methods at different pruning rates. Table 8 compares with the model sparsification training results in Figure 10B, where the accuracy loss of the experiment is controlled within 2% at the optimal pruning rate of 0.3. Furthermore, the method also provides better pruning results than other methods at a pruning rate of 0.4. Also, it shows that the impact of the pruning algorithm in the table on the accuracy of the YOLO V5s-CAcT network starts to increase significantly between the pruning rates of 0.3 and 0.4. The experimental results verify the effectiveness of the pruning method.

**Table 8:** Results of different pruning algorithms.

**Pruning**	**Model**	**PFD**	**Prune**	**Params (M)**
**algorithm**		**(mAp@0.5(%))**	**rate**	
FPGM-YOLOV5	YOLO V5s-CAcT3	89.06	0.3	4.18
		77.48	0.4	3.21
Slim-Filter -Pruner	YOLO V5s-CAcT3	87.68	0.3	3.99
		68.74	0.4	2.96
L1-Norm-Pruner	YOLO V5s-CAcT3	86.11	0.3	3.68
		44.99	0.4	2.57
L2-Norm-Pruner	YOLO V5s-CAcT3	86.54	0.3	3.82
		49.43	0.4	2.70

##### Model Distillation Results

Although the choice to use the optimal pruning rate maintains the accuracy of the model as much as possible, the accuracy of the detection is still considerably reduced compared to the original model. Using the knowledge distillation method, the model's accuracy can be restored, and the performance of the pruned model can even be further improved. This article divides the whole training process of knowledge distillation into two stages. First, the original model is selected as the teacher model, and four pruned models at a pruning rate of 0.4 are used as student models for the experiments. The student models are trained with different T(1, 5, 10, 15) by KD based on the effect of temperature T on model performance as proposed by Hinton's experiment (LeCun et al., 2015). The different pruning models were then trained with KD using the best-obtained temperature T. The training settings used the same hyperparameters as the previous experiments, but the optimizer used Adam and reduced the starting learning rate to 0.0001 and the α and β balance coefficients to 1.0 and 0.8, respectively.

**Figure 12:**
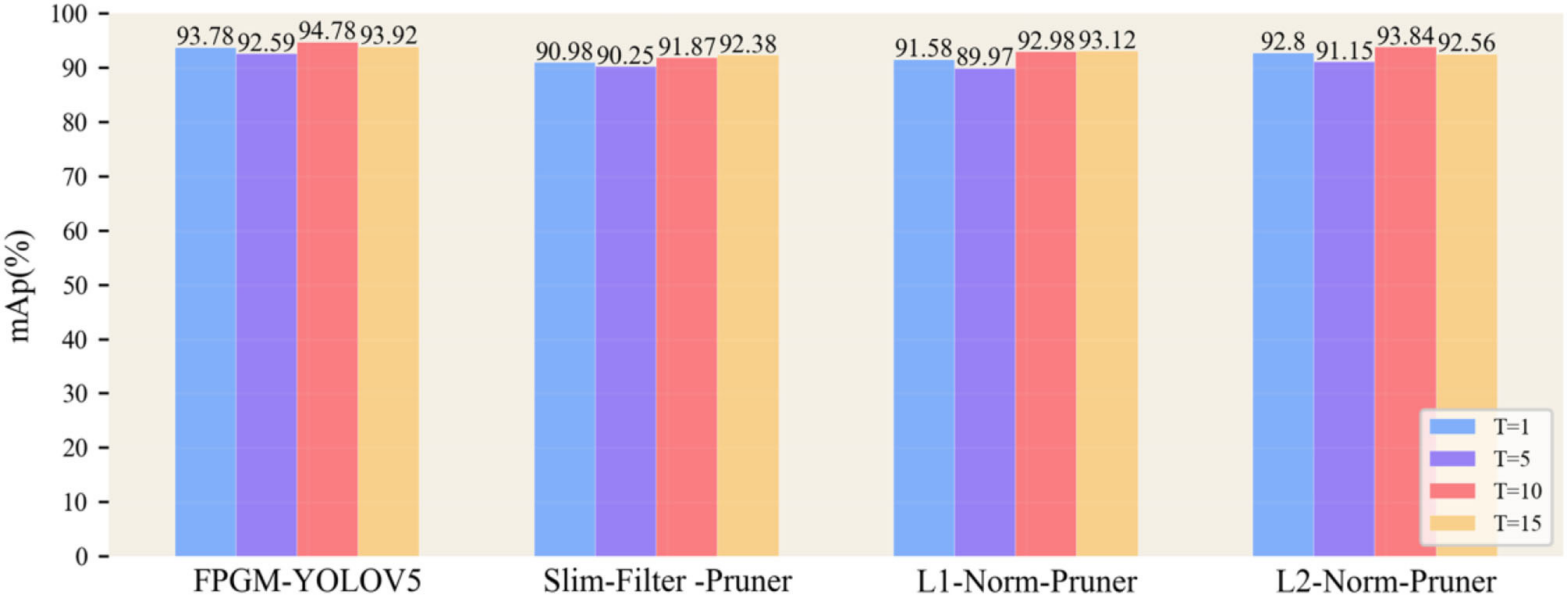
Effect of temperature parameters on knowledge distillation.

Using different temperatures T, with the same teacher model structure, the results are obtained as shown in Figure 12. Knowledge distillation improved model performance for the different pruning models, and the model size was smaller than the post-pruning model. For the four pruning models, distillation extraction was poor when T was 5, while at distillation temperatures T of 10 or 15, the models usually achieved better performance, close to complete accuracy.

Finally, the change in performance of the trimmed model after model distillation training is compared with the original model relative to the untrimmed model. As shown in Table 9, the average test accuracy, model parameters, and the amount of floating-point operations required to calculate the YOLO V5s-CAcT3 model for each of the four types of pruning algorithms. We also calculated the percentage change in performance of the knowledge distillation relative to the pruning model. The results show that the performance of the four pruned models has been dramatically improved by the method in this article, with a 56% reduction in the number of parameters and a 40% reduction in GFLOPs (Giga Floating-point Operations Per Second) compared to the original model, with slight change accuracy. The negative gain in the number of parameters and GFLOPs on the model performance is within 15.5%, significantly reduced if chosen a lower pruning rate, In the same case, the FPGM-YOLOV5 method proposed in this article showed no negative gain at more significant pruning rates but the improved model performance by 4%. Thus, the test results of the four types of pruning methods fully demonstrate that the model distillation technique solution in this article can significantly reduce the network parameters and significantly improve the operational efficiency of the model with less accuracy loss.

**Table 9:** Performance comparison of the original model, the trim rate 0.4 model, and the distillation temperature T of 15 models.

**Pruning algorithm**		**YOLO V5s-CAcT3 (Based on the YOLOV5s) in the PFD dataset**			
		**Original model**	**Pruned model**	**KD model**	**KD relative pruning change percent**
FPGM-YOLOV5	*mAp@*0.5 (%)	95.6	77.5	93.9	21.1
	Params (M)	6.8	3.2	3.1	−6.5
	GFLOPs	16.4	10.3	9.8	−4.9
Slim-Filter -Pruner	*mAp@*0.5 (%)	95.6	68.8	92.3	34.1
	Params	6.8	2.9	3.0	3.4
	GFLOPs	16.4	9.6	9.8	2.1
L1-Norm-Pruner	*mAp@*0.5 (%)	95.6	45.0	93.1	106.8
	Params	6.8	2.6	3.0	15.3
	GFLOPs	16.4	8.9	9.8	10.1
L2-Norm-Pruner	*mAp@*0.5 (%)	95.6	49.4	92.5	87.2
	Params	6.8	2.7	3.0	11.1
	GFLOPs	16.4	9.1	9.8	7.7

##### ActNN Model Results

Sparse training, pruning, and knowledge distillation of the model can effectively reduce the model parameters and improve the model performance, as the previous experiments using the YOLO V5s-CAcT3 model at various stages have fully confirmed. Due to hardware constraints, the model cannot be trained with limited GPU memory when the number of model parameters is large. The usual solution is to choose a smaller batch size or reduce the input image size, and in the limit case, only a model with a smaller number of parameters can be selected. This treatment will affect the convergence speed of the model and reduce its accuracy. Therefore, ActNN was chosen in this article to process the model, with YOLO V5m-CAcT and YOLO V5l-CAcT as the benchmark test models, and the dataset using PFD, with training settings and hyperparameters and improvement strategies configured according to the same attributes as YOLO V5s-CAcT3; Only the batch size and image size was changed during the training process. ActNN was turned on when GPU memory was insufficient, L3 was used for the compression level, each item was tested three times, and the average value was recorded at the end. The test results are shown in Table 10.

**Table 10:** ActNN compression test results at the L3 level.

**Model**	**Batch-size**	**Image-size**	**ActNN**	**mAp@0.5(%)**
YOLO V5m-CAcT	16	384	/	94.65
	32	384	/	94.84
	64	384	/	95.47
	64	512	/	OOM
	128	384	/	OOM
	64	512	L3	96.66
	128	384	L3	96.12
YOLO V5l-CAcT	4	384	/	94.64
	8	384	/	94.79
	16	384	/	95.35
	16	512	/	OOM
	32	384	/	OOM
	16	512	L3	96.89
	32	384	L3	95.97

In the two models tested with ActNN, the model's accuracy reached a high level without any significant accuracy drop. The smaller batch size corresponds to a slightly smaller accuracy caused by short training sessions. A larger batch size with the same number of training sessions can fix the model's accuracy and make it converge quickly, It also shows that using a small batch size does not improve model accuracy. YOLO V5l-CAcT has a more significant number of model parameters than YOLO V5m-CAcT, and ActNN compression of model parameters controls the model to train appropriately when both run out of memory. In terms of overall accuracy, the compressed parameter model has at least a 0.52% advantage over the YOLO V5s-CAcT3 model, with almost no loss of accuracy compared to the uncompressed YOLO V5m-CAcT model. In addition, the larger input image size has a higher model accuracy.

#### Performance Evaluation of Model Deployment

YOLO V5s-CAcT3 used YOLO V5s as the original model and achieved higher accuracy using the improved method proposed in this article. However, the running environment of the model, Python, limits its development in the agricultural field and increases the operational costs. Therefore, this article optimizes the process by proposing two technical routes to compress the model. Technical route 1 complements technical route 2. The trained deep neural network model is compressed, followed by further optimization using simplified operators with INT8 quantization, and deployed in the NCNN framework to maximize detection performance. The experimental results of the training and model deployment are described in Table 11. The tests were performed using the PFD dataset and compressed with ActNN to YOLO V5s-CAcT3. The speed in Table 11 is the average inference time per image in the test dataset.

**Table 11:** Deployment performance results.

**Model**	**Methods**	**Model size (MB)**	**mAp@0.5(%)**	**Speed (ms)**
YOLO V5s-CAcT3	Model training and selection	13.952	95.624	3.589
	FPGM-YOLOV5 pruning	9.723	89.062	3.254
	Knowledge distillation	6.311	93.983	3.452
	Simplify operator	6.158	93.594	3.236
	INT8 export	1.782	93.285	2.173
	NCNN deployment	1.835	94.237	1.430

As can be seen from the experimental results, after a series of deployment optimizations, the model deployed in NCNN was reduced by 88%, the inference time was reduced by 72%, and the model *mAp@*0.5 fluctuated by no more than 1.5%. A single compression scheme has a compression ratio of over 50%, and multiple compression schemes can be used. For complex PFD datasets, the method achieves a speed-up of 2.2–2.5 times in YOLO V5s-CAcT3, based on the strategy in this article. This demonstrates the feasibility and effectiveness of NCNN as a deployment solution. The various phases proposed in this article have a highly consistent articulation, and this approach brings convenience to development and deployment.

## Conclusion

This work will encourage future research into alternative deep learning models tailored to specific application tasks. The technical routes proposed in the current research are complementary, meeting the training requirements, testing and deploying models under different hardware conditions, and providing flexibility for researchers to choose from.

This study proposes a fast, efficient, and broadly applicable solution for industrial-scale crop disease image detection tasks for crop leaf diseases and uses various model compression techniques to improve model performance. The PFD dataset was first constructed using PlantVillage with PLD to replace erroneous disease categories in the AI Challenger dataset, and multiple data enhancement methods were used to balance the smaller data samples. The adaptive image enhancement approach RA strategy is then used to improve the ability of the original YOLO V5 model to learn invariant features. Because the sample may contain an imbalance of positive and negative samples, the original model loss function is altered using the FocalLoss and SmoothBCE technique to reduce the risk of model overfitting and improve model robustness. In addition, to improve the convergence ability of the model and reduce the model training time, we integrated the Early Stopping mechanism into the model. These several techniques collaborated to improve the original YOLO V5 model and formed the YOLO V5s-CAcT3 model structure, which realized the disease detection of various crop leaf images. The effects of model storage size, inference time, deployment cost, and application environment on the model in agricultural disease application scenarios are also considered. The proposed FPGM-YOLOV5 pruning method achieves significant results on YOLO V5s-CAcT3, and the proposed method outperforms other pruning methods. Later, the performance of the pruned model was restored using the knowledge distillation technique, and better results were achieved at different temperatures T in the same test environment, and the performance was close to that of the original model. In addition, the proposed ActNN performs activation parameter compression on the training model, which solves the problem of poor hardware performance or training large parameter models. Finally, the model performance is improved further with the help of simplified operators and INT8 quantization on the model, and the best results are obtained by deploying the model in NCNN with an 88% reduction in model size and a 72% reduction in inference time compared to the original method, saving a significant amount of computational cost and time. The findings show that the current state-of-the-art ActNN, YOLO V5, and mutual collaboration of model pruning and knowledge distillation techniques have all achieved better results, effectively solving common problems in current agricultural disease image detection, and have broad application prospects for precision agriculture and agricultural industry efficiency. Research in this area will be expanded in the future to include more complex farming scenarios.

## Data Availability Statement

The original contributions presented in the study are included in the supplementary materials, further inquiries can be directed to the corresponding authors.

## Author Contributions

GD contributed to conceptualization, data curation, methodology, software, validation, visualization, investigation, writing the original draft, project administration and writing, reviewing, and editing the manuscript. JF contributed to funding acquisition, resources, and supervision. All authors have read and agreed to the published version of the manuscript. All authors contributed to the article and approved the submitted version.

## Funding

This work was supported by the Inner Mongolia Autonomous Region Science and Technology Major Special Project: R&D and demonstration of critical technologies for intelligent control of the potato industry chain (2021SZD0026).

## Conflict of Interest

The authors declare that the research was conducted in the absence of any commercial or financial relationships that could be construed as a potential conflict of interest.

## Publisher's Note

All claims expressed in this article are solely those of the authors and do not necessarily represent those of their affiliated organizations, or those of the publisher, the editors and the reviewers. Any product that may be evaluated in this article, or claim that may be made by its manufacturer, is not guaranteed or endorsed by the publisher.

## Figures and Tables

**Figure A1:**
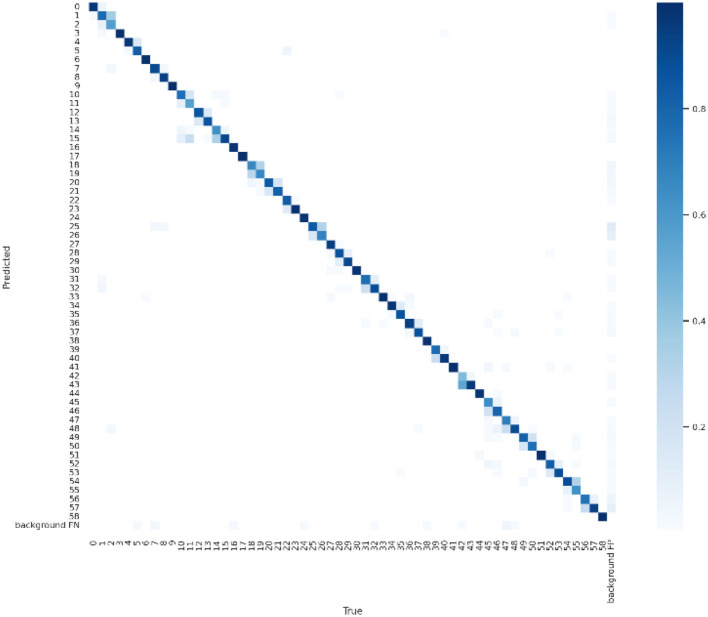
Confusion matrix for the predictions of the best model trained on the PFD dataset.

**Figure A2:**
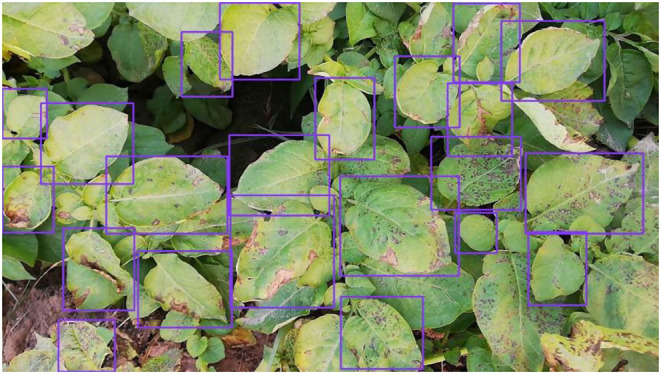
Prediction of the best model trained on the PFD dataset and live labeling of the sample images.

**Figure A3:**
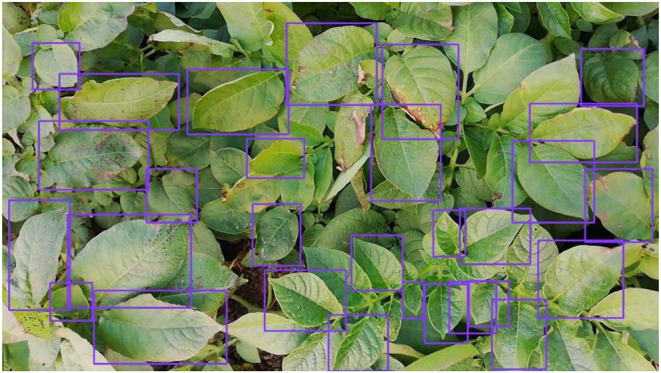
Prediction of the best model trained on the PFD dataset and live labeling of the sample images.
